# Comparison of methods for *in vivo* and *ex vivo* quantification of iron in murine brain tumors

**DOI:** 10.1371/journal.pone.0356378

**Published:** 2026-08-17

**Authors:** Jessica L. Veenstra, Sidney A. Schwartz, Joseph P. Speth, Justin B. Geise, Thomas R. Grier, Chandler J. Zaugg, Zhen Jiang, Justina J. Riffell, Teagan P. Ames-Majeski, Aaron J. Specht, Matthew L. Scarpelli

**Affiliations:** 1 School of Health Sciences, Purdue University, West Lafayette, Indiana, United States of America; 2 Purdue University Interdisciplinary Life Sciences Program (PULSe), West Lafayette, Indiana, United States of America; VIT University, INDIA

## Abstract

**Background:**

Iron is a redox-active biological trace element with concentration-dependent effects on human health and disease. This study evaluated multiple methods to quantify iron oxide nanoparticles for potential theragnostic applications in brain cancer.

**Methods:**

Iron quantification protocols were developed for magnetic resonance imaging (MRI), x-ray fluorescence spectroscopy (XRF), UV-visible spectroscopy (UV-Vis), and inductively coupled plasma mass spectrometry (ICP-MS). The techniques were evaluated in cell culture models and murine brain tumors treated with the iron oxide nanoparticle ferumoxytol. All methods were assessed for correlation between administered ferumoxytol dose and iron measurement as well as agreement with the ICP-MS gold standard.

**Results:**

In vivo MRI T2* and XRF iron measurements correlated with administered ferumoxytol dose (T2*: ρ = −0.64, p = 0.0034; XRF: ρ = 0.97, p = 2 x 10^-15^) and ex vivo ICP-MS results (T2*: ρ = −0.69, p = 0.012; XRF: ρ = 0.91, p < 2.2 x 10^-16^) with stronger, more significant correlations emerging for XRF than MRI T2*. However, unlike MRI, XRF cannot identify the spatial distribution of iron deposits. The developed UV-Vis protocol quantified iron in cell culture samples but failed in ex vivo brain tumor tissue.

**Conclusion:**

The successful in vivo and ex vivo quantification of tumor iron accumulation indicates that these methods are relevant in preclinical and clinical settings involving diagnosis and treatment of various human diseases. This work establishes a clear framework for choosing the ideal method based on iron concentration, equipment, budget, and sample identity.

## Introduction

Iron is a trace biological element with high redox activity that plays a vital role in maintaining human health. It serves as a necessary component for biological cofactors involved in oxygen transport, energy production, and DNA replication and repair [1–6]. However, iron also plays a role in the generation of reactive oxygen species which can damage cellular structures, induce genetic mutations, encourage inflammation, and contribute to cell death [1,7]. The wide-ranging effects of iron on biological functions can combat or contribute to a variety of disease states [8–18]. Iron chelators and iron replacement therapies both have applications in the treatment of a variety of diseases, making iron-based therapies an interesting area of study, particularly in diseases such as cancer [19,20]. Furthermore, the concentration of iron in select organs in the body can be a predictor of disease severity or certain treatment responses [21–26].

Methods to quantify iron are invaluable tools in preventive care, diagnosis, and even treatment for a variety of diseases. In this study, multiple methods for *in vivo*, *ex vivo,* and *in vitro* iron quantification are assessed to provide novel side-by-side evaluation of diverse methods for iron quantification. These methods are applied in a murine glioblastoma model or glioma cell cultures after administration of an iron oxide nanoparticle, ferumoxytol. Ferumoxytol is an FDA-approved therapy for patients with iron deficiency anemia, and it is also used off-label as an MRI contrast agent as well as an experimental cancer therapy [27]. As of 2019, the manufacturer reported over 2.6 million patient exposures to ferumoxytol, but data regarding incidence of its off-label use as a contrast agent is more difficult to find [28]. However, a large study published in 2019 conglomerating data from 11 medical centers over a 15-year period recorded 4,240 uses of ferumoxytol as an MRI contrast agent in 3,215 patients [29]. The application of ferumoxytol in therapeutic and diagnostic settings indicates potential theragnostic applications for this drug where it is used to both treat and monitor diseases. Quantification methods for ferumoxytol are thus important to determine potential therapeutic concentrations and assess disease progression.

The methods evaluated for quantifying ferumoxytol iron include T2* magnetic resonance imaging (MRI), x-ray fluorescence spectroscopy (XRF), ultraviolet-visible (UV-Vis) spectroscopy, and inductively coupled plasma mass spectrometry (ICP-MS). More established methods for iron quantification (MRI and ICP-MS) are compared to more accessible and inexpensive methods (XRF and UV-Vis spectroscopy). A murine glioblastoma mouse model receiving ferumoxytol treatment is well suited for this research as ferumoxytol is known to have tumor-specific accumulation as well as theragnostic potential [30]. Furthermore, the brain has low endogenous iron levels and is well-separated from organs in the body cavity with high endogenous iron levels or iron filtration functions, providing low background levels of iron [31] These characteristics produce an ideal system to develop, test, and compare methods for *in vivo* iron quantification.

MRI is the most clinically relevant of the assessed methods for iron quantification and is often preferred in clinical settings over biopsy-related methods for its noninvasive nature. It has been accepted by many as a de facto gold-standard method for noninvasive quantification of iron levels throughout the body [32]. MRI detects the signal from proton magnetization following radiofrequency excitation. Following this excitation, T2 is a measure of the intrinsic transverse (spin-spin) relaxation of protons leading to loss of phase coherence, and T2* is a measure that incorporates T2 but also measures the effects of local inhomogeneities in the magnetic field on transverse relaxation [33]. The magnetic properties of iron accelerate transverse proton dephasing, shortening T2 and T2* and causing regions with high concentrations of iron to appear darker on T2- and T2*-weighted images. Additionally, iron increases local magnetic field inhomogeneities in a concentration-dependent manner, further shortening T2. Iron concentration thus has a more prominent effect on T2* than T2, and T2* is more sensitive to changes in iron concentration and more widely used for iron quantification than T2 alone [34]. A quantitative estimation of T2* values for any given tissue is obtained by fitting an equation to a time series of the correct type of MR images [33]. The accuracy of this method has been confirmed in the brain by a study that found that R2* (the inverse of T2*) for *in situ* post-mortem brain scans in humans strongly correlated with iron concentrations obtained using ICP-MS after tissue isolation [34].

XRF is a lesser-known method with limited clinical utilization [35–38]. X-rays are used to excite electrons in a sample, and the following relaxation of electrons leads to the emission of a unique signature of photons for each element, the intensity of which is dependent on the concentration of that element [39]. A subset of XRF devices are portable, and although they are slightly less sensitive than benchtop XRF models, this portability provides a level of convenience and flexibility which is not available with any of the other assessed methods for iron quantification.

UV-Vis spectroscopy is a basic method for iron quantification that relies on the dissolution of the sample and the conversion of all the iron into a single chemical compound such as FeCl_3_, the concentration of which can be identified by measuring the absorbance of the sample at the known maximum absorbance wavelength for that compound [40]. This method has been previously used for determining the concentration of iron nanoparticles and nanobiocomposites in basic solutions as it is able to overcome unique challenges related to nanoparticle dissolution [40–42]. These protocols are highly accessible as only common reagents and basic lab instruments are required.

ICP-MS has long been considered the gold standard for quantifying many elements, including iron, in solution. In clinical settings, this requires a biopsy, which is invasive and measures iron content only in a small portion of the desired organ, tumor, or other feature [43]. However, ICP-MS is considered a superior method for iron quantification due to its low limit of detection which enables highly accurate results for concentrations of trace elements like iron [44]. With this technique, liquid samples are atomized and ionized by high-temperature argon plasma and then extracted into a quadrupole mass analyzer which separates ions based on their mass-charge ratio for detection and identification.

This work fills a significant research gap by assessing all four of these methods for iron quantification, providing other researchers with a practical framework to determine the optimal method for iron quantification on a situational basis. Furthermore, inclusion of detailed methodology allows for easy replication of these methods.

## Results

### Results for living organisms

#### MRI.

To assess the sensitivity and linearity of T2* maps for iron detection, 4.2% agarose phantoms were prepared with concentrations ranging from 0 to 5000 ppm of ferumoxytol to generate a calibration curve (S1A Fig). For this method, linear results were obtained for median T2* values in phantoms ranging in concentration from 0 to 250 ppm of iron (S1B Fig). Phantoms with 750 ppm or more had extremely high median T2* values with voxel-wise standard deviations in excess of two orders of magnitude larger than the median values, producing an incorrect positive trend, confirming difficulty in quantifying very high levels of iron with this method. Using the standards between 0 and 250 ppm of iron produced the correct negative trend (y = −0.024x + 6.7, *ρ* = −1, *p* = 0.33). The estimated limit of detection for T2* in these phantoms was 180 ppm of iron.

**Fig 1:**
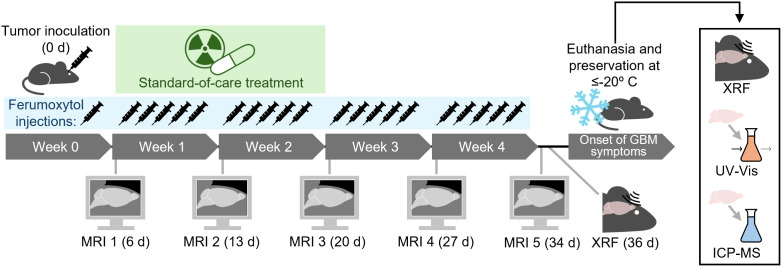
Murine study layout. Design of murine study with standard-of-care chemoradiotherapy and ferumoxytol treatments, MRI and XRF monitoring, and postmortem XRF, UV-Vis spectroscopy, and ICP-MS. Schematic assembled in PowerPoint using icons sketched in Inkscape.

For the *in vivo* assessment, mice were intracranially inoculated with GL-261 glioma cells and subjected to a treatment plan involving a standard-of-care chemoradiotherapy regimen and 21 ferumoxytol injections ranging in dose from 0 to 300 mg/kg/day (Fig 1). This dose range notably exceeds the clinically relevant dose of 510 mg total [45]. However, attempts to mimic this dose in mice receiving 6 mg/kg/day resulted in no measurable infiltration of ferumoxytol (data not published). As the plasma half-life of ferumoxytol is much shorter in rodents (<4 h) than in humans (12–14 h), we utilized a surface area dose conversion based on guidance from the FDA, which helps to account for size-related changes in metabolism. [46–50]. Using this conversion, the human equivalent dose for the 20 mg/kg dose in mice is 1.6 mg/kg, and the human equivalent dose for the 300 mg/kg dose in mice is 24.3 mg/kg. This dose range contains the clinically relevant dosage. It is also important to note that doses higher than the current clinically relevant range for iron deficiency anemia may be necessary for the application of ferumoxytol in experimental cancer therapies. Actual dose rather than human equivalent dose is reported throughout for simplicity.

**Fig 2:**
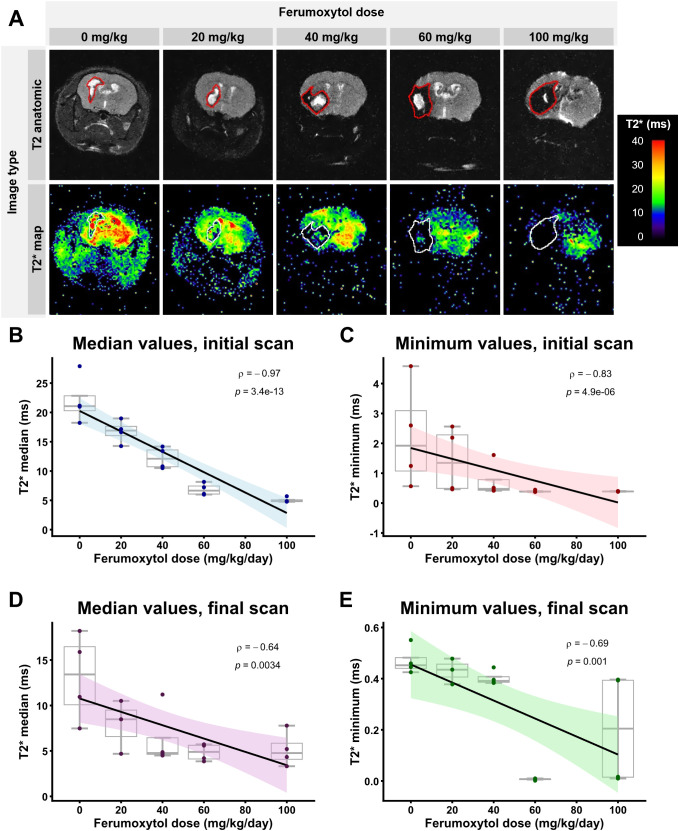
Use of MRI for the quantification of iron in live mice. (A) T2 anatomic images and co-registered T2* maps of representative murine brain scans at specified doses on day 34 post tumor inoculation (final scan). Tumors are outlined in red or white. (B) Median T2* values and (C) minimum T2* values calculated for the tumor region 6 days post tumor inoculation (1 ferumoxytol dose previously given). (D) Median T2* values and (E) minimum T2* values calculated for tumor region 34 days post tumor inoculation (21 ferumoxytol doses previously given). For all plots, N = 3-4 mice per treatment group. Lines indicate the best fit linear regression, and the shaded regions represent the 95% confidence interval for the regression.

Initial analysis of iron infiltration into the brain was conducted with MRI. Qualitatively, increases in size and intensity of hypointense regions on T2 anatomic images and T2* maps were visually apparent in the tumor, indicating increased iron accumulation (Fig 2A, S2-S6 Figs). However, images from the mice receiving doses higher than 100 mg/kg/day of ferumoxytol were uninterpretable as significant distortions and artifacts were present, which thus limits the ability of iron to be quantified with standard methods for MRI at high concentrations (S7A-B Fig).

Iron accumulation was quantified using T2* values within the identified tumor regions in mice receiving doses up to 100 mg/kg/day of ferumoxytol. For the mice treated with ferumoxytol doses between 0 and 100 mg/kg/day, there was a strong inverse correlation between median T2* and administered ferumoxytol dose. This relationship was strongest on the initial MRI (ρ = −0.97, p = 3.4 × 10^−13^) after the mice had received only one dose of ferumoxytol (Fig 2B), but was maintained throughout the course of treatment, continuing to the last MRI (ρ = −0.64, p = 0.0034) 3 days after the conclusion of treatment (Fig 2D). A similar trend was observed when comparing minimum T2* and ferumoxytol dose (initial scan: ρ = −0.83, p = 4.9 × 10^−6^; final scan: ρ = −0.69, p = 0.001), with the limitation that on the final MRI scan, T2* minimum values for the 100 mg/kg/day dose became erratic with higher variation between mice than was observed at earlier timepoints, likely due to the substantial magnitude of iron accumulation over time (Fig 2C, 2E).

Analysis of all MRI timepoints utilizing the T2* median and minimum revealed statistically significant decreases in values over time, capturing the temporal accumulation of iron (S8A-B Fig). A downward but nonsignificant trend was also observed in mice receiving 0 mg/kg ferumoxytol, likely due to changes in the morphology of the tumor over time. This source of variation may indicate that T2* is a better measure of iron in early-stage tumors than late-stage tumors or tumors that have undergone treatments, potentially due to the more varied morphology of the latter two conditions.

#### Portable XRF.

Due to limitations encountered with MRI at high iron concentrations, XRF was used as a secondary method for *in vivo* iron quantification. A calibration curve was generated with this method to assess the sensitivity and linearity (S9a-b Fig). Phantoms between 0 and 1250 ppm produced a linear result (y = 0.012x – 0.3, *ρ* = 1, *p* = 0.017). The estimated limit of detection for pXRF in these phantoms was 156 ppm of iron.

**Fig 3:**
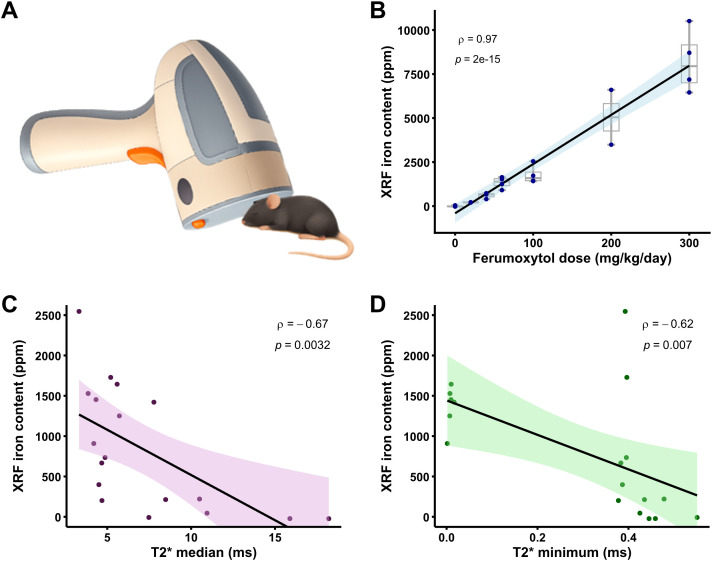
Use of pXRF for the quantification of iron in live mice. (A) Illustration of pXRF setup for detection of tumor infiltrating iron in mice. Created in Illustrae. (B) Correlation between pXRF iron content and ferumoxytol dose. N = 2-4 biological replicates per treatment group. (C) Correlation between pXRF iron content from day 36 and T2* median values from day 34 MRI in individual mice. N = 18 total biological replicates. (D) Correlation between pXRF iron content from day 36 and T2* minimum values from day 34 in individual mice. N = 14 mice total. For all plots, lines indicate the best fit linear regression, and the shaded regions represent the 95% confidence interval for the regression.

For *in vivo* assessment, mice were anesthetized and held within the beam of a pXRF analyzer for 30–60 seconds, with the top of their skull positioned so that the center of the beam was passing through the right hemisphere of the brain where the tumor had been implanted (Fig 3A). For all mice, there was a strong correlation between iron concentration measured by pXRF and ferumoxytol dose administered up to the highest dose of 300 mg/kg/day (Fig 3B). MRI results and pXRF results collected three days apart shortly after the conclusion of treatment in the mice showed moderate to strong correlation when comparing pXRF results to T2* median or T2* minimum (Fig 3C-3D).

These results indicate that pXRF is a quick and convenient method to quantitively measure iron accumulation *in vivo* in murine brain tumors, even when iron concentrations are very high. However, for mice receiving 0 mg/kg/day of ferumoxytol, the mean value for iron concentration measured by XRF was essentially zero (−1.8 ppm) despite low levels of endogenous iron in the brain tissue. This indicates potential signal attenuation from the skull and surrounding tissue or a matrix mismatch between the calibration settings and this application, which would result in a systematic offset of the values not affecting the dose-response relationship.

### Results for tissue samples or postmortem organisms/tissue

#### Comparison of XRF and UV-Vis spectroscopy in tissue culture experiment.

To further explore potential methods for the quantification of iron, methods that could be used postmortem or on *ex vivo* samples were also tested and compared to *in vivo* methods. These methods include ICP-MS, UV-Vis spectroscopy, and XRF. While ICP-MS has already been established as a gold standard method for quantifications of nearly all elements in a sample, UV-Vis spectroscopy and XRF are less established methods for complex biological samples. In order to first develop protocols and determine lower limits of detection for these methods, basic tissue culture experiments were utilized.

**Fig 4:**
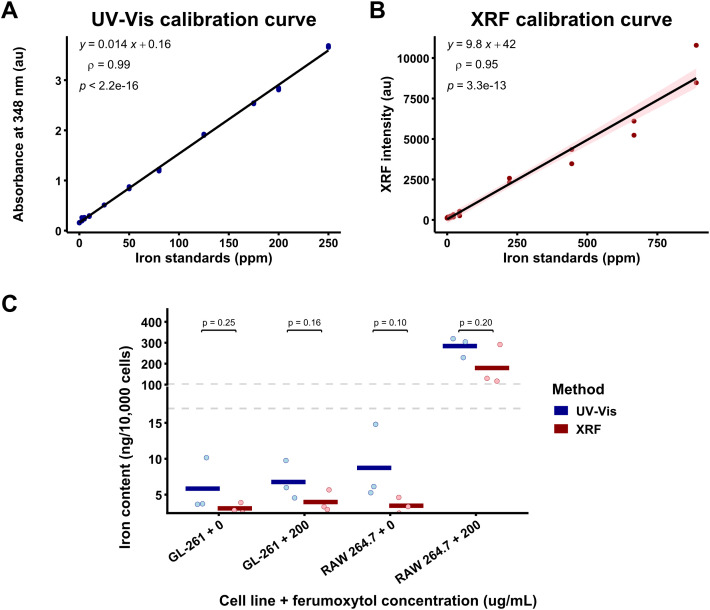
Comparison of UV-Vis spectroscopy and bXRF protocols for quantification of iron concentrations. (A) Calibration curve generated with UV-Vis spectroscopy protocol. N = 4 replicates per dose. (B) Calibration curve generated with bXRF protocol. N = 2-3 replicates per dose. For plots 4a-b, lines indicate the best fit linear regression, and the shaded regions represent the 95% confidence interval for the regression. (C) Cell culture assay testing the uptake of ferumoxytol in GL-261 and RAW 264.7 cell lines over a 48-hour period. Mean line is shown with points representing independent biological replicates. N = 3 biological replicates. Logarithm transformed Student’s t-test p-values are shown.

Ferumoxytol standards were prepared for both methods to generate a standard curve for the determination of iron concentrations. The UV-Vis spectroscopy method requires that samples be dissolved in HCl and diluted to a final concentration of 4.9 M HCl to ensure all iron is detectable as FeCl_3_. Although bXRF analysis can be performed on intact cell samples, the bXRF samples were also dissolved in HCl with a final concentration of 4.9 M in order to make more direct comparisons between the two methods. Absorbance spectra for the UV-Visible spectroscopy standards were then used to confirm the maximum absorbance of FeCl_3_ occurred at 348 nm (S10A Fig). The standard curve for UV-Vis spectroscopy using absorbance values at 348 nm was highly linear (y = 0.014x + 0.16, *ρ* = 0.99, *p* < 2.2 x 10^-16^) with a calculated lower limit of detection of 9.35 ppm of iron (Fig 4A). The standard curve for benchtop XRF (bXRF) was also highly linear (y = 9.8x + 42, *ρ* = 0.95, *p* = 3.3 x 10^-13^), but with a higher calculated lower limit of detection than UV-Vis spectroscopy at 169 ppm (Fig 4B). Because the HCl dissolution step was not required for bXRF analysis, standard concentrations post dilution were used for the bXRF calibration curve and limit of detection calculation.

Samples of the GL-261 glioma cell line and the RAW 264.7 macrophage cell line, which had been cultured with and without ferumoxytol, were divided between the two methods for direct comparison of quantified iron levels (Fig 4c). UV-Vis and XRF measurements for each individual condition revealed similar results between the two methods. Although UV-Vis spectroscopy readings trended higher than bXRF readings, no significant differences were observed. Previous ICP-MS results from a similar study by Feng et al. were used to confirm the obtained values were reasonable [17]. This study found limited ferumoxytol uptake by the GL-261 cell line, but significant uptake by the RAW 264.7 line, further confirming that macrophages have a high propensity for ferumoxytol uptake.

### Utilizing XRF and UV-Vis spectroscopy on mice postmortem

**Fig 5:**
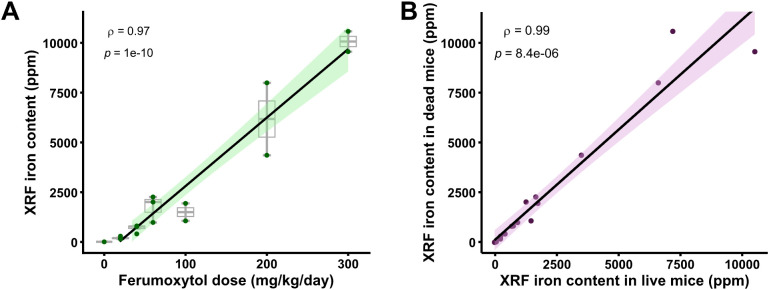
Use of pXRF for the quantification of iron in postmortem mice. (A) Correlation between XRF iron content and ferumoxytol dose. N = 2-4 biological replicates per treatment group. (B) Correlation between live and postmortem pXRF iron content readings in individual mice. N = 19 total biological replicates. Lines indicate the best fit linear regression, and the shaded regions represent the 95% confidence interval for the regression.

Postmortem pXRF once again revealed a strong linear relationship between measured iron content and ferumoxytol dose (Fig 5a). Postmortem results were similar to pXRF results obtained while the mice were still alive, indicating there was likely little change in iron content between the conclusion of treatment and the onset of symptoms, which was an average of 35 days (Fig 5b). In addition, these results also indicate that pXRF may still be used on postmortem tissue which has been thawed after storage at ≤ −20º C.

Unfortunately, UV-Vis spectroscopy proved to be a difficult method to transition from use on a single cell suspension to solid tissue samples. This method relies on the dissolution of the sample in concentrated hydrochloric acid, but the brain tissue proved to be too complex of a biological sample for complete dissolution by this method. Absorbance readings were highly affected by inhomogeneities and solids remaining in the sample, and thus this method initially provided no interpretable results.

Further attempts to adapt the UV-Vis spectroscopy protocol involved processing tumor samples into single cell suspensions immediately after euthanasia, followed by dissolution in concentrated hydrochloric acid and analysis. However, absorbance readings still deviated from the expected values with unidentified compounds contributing to absorbance values at a variety of wavelengths, making the maximum absorbance peak for FeCl_3_ unidentifiable (S10B Fig). These results indicate limitations of this method for the assessment of complex biological samples.

### Utilizing ICP-MS on mice postmortem

**Fig 6:**
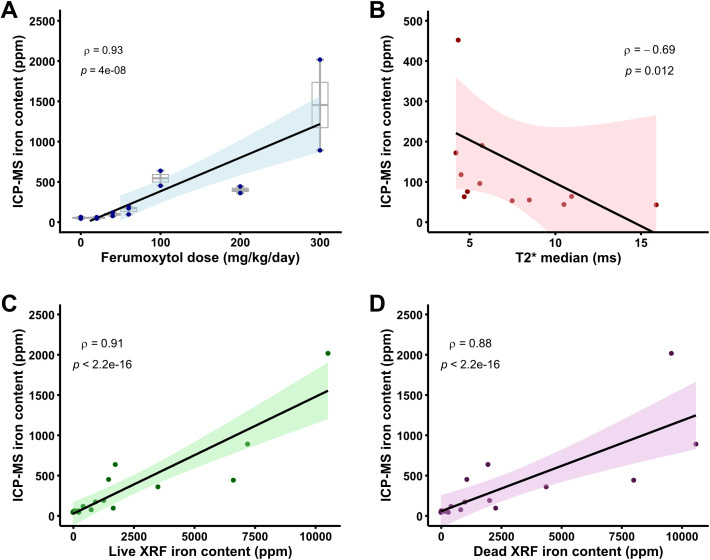
ICP-MS results and comparison with all iron quantification methods tested on live or postmortem mice. (A) Correlation between ICP-MS iron content of postmortem brain tissue and ferumoxytol dose. N = 2 biological replicates per treatment group. (B) Correlation between ICP-MS iron content of postmortem brain tissue and T2* median values on day 34 for individual mice. (C) Correlation between ICP-MS iron content of postmortem brain tissue and pXRF iron content obtained on day 36 from live mice. (D) Correlation between ICP-MS iron content of postmortem brain tissue and pXRF iron content of postmortem brain tissue measures in situ. For B through D, N = 14 total biological replicates. For all plots, lines indicate the best fit linear regression, and the shaded regions represent the 95% confidence interval for the regression.

ICP-MS was the final method for iron quantification that was tested. Similarly to the other methods, a strong linear relationship was observed between total iron content and ferumoxytol dose (Fig 6A).

### Comparisons with ICP-MS

As ICP-MS is considered the gold-standard method for iron quantification, all other methods were compared to this method. A moderate significant negative trend (Fig 6B, *ρ* = −0.69, *p* = 0.012) was observed between T2* median from the final MRI scan and ICP-MS results. ICP-MS results correlated more strongly with pXRF *in vivo* measurements (Fig 6C, *ρ* = 0.91, *p* < 2.2 x 10^-16^) and pXRF post-mortem measurements (Fig 6D, *ρ* = 0.88, *p* < 2.2 x 10^-16^). The observed parts per million for ICP-MS were typically between 10–30% of the observed parts per million for pXRF measurements, but this difference is likely explained by the use of the whole brain for ICP-MS analysis, whereas the XRF analysis focused on the tumor region, where site-specific accumulation of iron was occurring. Based on the MRI data, the lethal tumor size was, on average, greater than 15% of total brain volume (> 60 mm^3^), which further confirms the lower values observed in ICP-MS are appropriate when compared to the pXRF readings. The observed strong correlation indicates that both methods are effective for measuring iron concentrations.

## Discussion

Several studies already exist establishing positive correlations between ICP-MS and T2* (or R2*), ICP-MS and XRF, or ICP-MS and UV-Vis spectroscopy [34,35,40]. In addition to confirming these findings, this is the first study to bring all four methods together for use and comparison on the same set of samples.

In this study, multiple well-established and lesser-used methods for quantifying iron in biological samples were tested and compared. Protocols allowing for the accurate and reproducible use of each method were developed and optimized. MRI T2* and pXRF were established as reasonable methods for quantifying tissue iron levels in living organisms. pXRF and ICP-MS were used to quantify iron in tissue samples from postmortem mice. In more basic biological samples like single cell suspensions, both bXRF and UV-Vis spectroscopy were effective.

ICP-MS, the overarching gold standard method, acted as a control and comparison for the other iron quantification methods. This method is widely used and highly accepted as accurate, but it is less than ideal in many settings for several reasons. These reasons include the high cost of equipment and reagents, lengthy and destructive sample preparation, potential for nonrepresentative biopsy samples, and inability to use this method for noninvasive *in vivo* analysis.

Median and minimum T2* values from *in vivo* MRI were shown to correlate strongly with ferumoxytol dose and moderately with *ex vivo* ICP-MS measurements. The exact cause of the limited strength of this correlation is unclear but may be due to several known limitations of this method. Perhaps most notably, many other factors influence T2*. These can include tissue oxygenation, hemorrhage, and calcification [51,52]. While the impacts of oxygenation and hemorrhage on T2* are in part due to the proteins containing endogenous iron involved in these processes, these tissue states and others like calcification represent additional sources of variation for magnetic susceptibility that are instead likely driven by other tissue components or structures [53]. Furthermore, the oxidation state of iron has been shown to significantly impact the relaxation rate measured by MRI [54,55]. These factors may have played an increasing role as the study progressed, as T2* trended down in the control mice over time and the correlation with ferumoxytol dose weakened over time across the dose range. Future work could assess the progression of hypoxia, hemorrhage, or extracellular matrix formation in these tumors and the effects of these factors on T2*. Another drawback of T2*-weighted MRI in this experiment was its relatively low upper quantifiable limit for iron concentration. Ferumoxytol doses exceeding 100 mg/kg/day produced local magnetic field inhomogeneities of sufficient magnitude to cause rapid signal dropout and image distortion that prevented accurate quantification. This occurs when magnetic field inhomogeneities accelerate transverse proton dephasing, causing the T2*-weighted signal to decay below detectable levels before the first echo is acquired. The 7 T MRI field strength may have played a limiting role as there is an inverse relationship between field strength and T2* [56]. However, comparison of the T2* values of healthy versus tumor tissue or tumor tissue over a series of time points can still provide important information about relative amounts of iron accumulation up to the amount observed in the 100 mg/kg/day dosing group, especially in instance comparable to this study where treatment with ferumoxytol led to significant accumulation of iron.

XRF is a lesser-known method but proved to have merit as *in vivo* pXRF measurements correlated well with ferumoxytol dosage, as well as correlating moderately with *in vivo* MRI T2* and strongly with *ex vivo* ICP-MS results. As this method lacks image guidance and the ability to precisely isolate the region of interest, potential limitations include incorrect beam placement and measurement bias introduced by iron accumulation in superficial tissues such as skin and hair. The small beam size does facilitate placement within the target region of interest, and superficial tissue structures are expected to account for a minimal proportion of the total tissue volume sampled. However, their contribution to measurement output cannot be excluded. It is also important to note that although quantification of brain iron concentrations was possible in mice with pXRF, these measurements may not be possible for a human brain due to significantly increased thickness of the skull, which attenuates the XRF signal. However, element levels in other more superficial organs or bones have been successfully quantified with pXRF in humans [35–38]. Overall, this method is a cost-effective way to quantify iron in tissue culture samples, tissue samples, and animals in preclinical studies. It also has potential as a cost effective, convenient, and noninvasive way to measure iron *in vivo* in humans, and this is an area of future study. Further optimization of the established protocol for quantification of lower iron concentrations would further enhance this method.

Determining the true lower and upper LOD for either of the *in vivo* methods is difficult as varying results were seen in the phantom data and the *in vivo* data, and this could be due to several reasons. The phantoms fail to capture the biological complexity and associated inhomogeneity in iron distribution which could affect outputs, especially for a spatial method such as T2* mapping. Biological processes shifting the oxidation state of iron or incorporating it into different transport or storage proteins will impact its magnetic properties and overall distribution, influencing MRI readouts [54,55]. The incorporation of iron into proteins and tissue in general will create different XRF attenuation patterns and altered photon paths for excitation and fluorescence than agarose alone [57]. The internal pXRF calibration used in the *in vivo* studies was not created with standards which replicated murine tissue structure, and thus this could be an additional source of variation which creates significant differences between the *in vivo* and phantom measurements. These factors limit the applicability of LOD calculations from the phantoms to the *in vivo* data and may explain differences in apparent concentration ranges with linearity between the phantoms and *in vivo* data. However, both the phantom and *in vivo* data indicate concentration ranges with high linearity for the relationship between iron concentration and T2* or XRF output, strengthening the evidence that these methods are suitable for iron quantification and indicating that effective LOD can vary when assessing iron concentrations in different settings.

Multiple UV-Vis spectroscopy protocols exist for the quantification of iron [40–42]. These protocols have been shown to be highly accurate and reproducible in simple biological samples. However, some of these protocols are limited in their ability to measure iron nanoparticles as they are designed for the quantification of free iron. Other protocols have limited compatibility with complex biological samples. In this paper, an adapted protocol was established that allowed for the dissolution of iron nanoparticles and removal of undissolved debris in sample from cell culture. In cell culture samples or less complex samples, UV-Vis proved to be highly accessible and cost effective as it requires only a few basic reagents and a plate reader capable of measuring absorbance at 348 nm. However, this protocol was not successfully adapted for use with more complex tissue samples. Furthermore, this method is destructive, meaning that it cannot be used in living organisms and samples cannot be retained in their original form for further research. Altogether, this work showed that ICP-MS, MRI, XRF, and UV-Vis spectroscopy can all be used for the quantification of iron. The selection of the most ideal method for a particular project is highly dependent upon the state of the sample, whether or not the sample must be retained for other analyses, and what equipment is available (Table 1).

**Table 1:** Comparison of methods.

	Use in live subjects*	Non-destructive	Equipment cost	Cost per run	Specificity to ROI**	Specificity to iron	LOD***
**MRI**	Yes	Yes	$$$	$$$	High	Moderate	High lower limit, low upper limit
**pXRF**	Yes, with limitations	Yes	$$	$	Limited	High	High lower limit
**bXRF**	No	Yes	$$	$	High	High	High lower limit
**UV-Vis**	No	No	$	$	High	High	Good
**ICP-MS**	No	No	$$	$$$	High	High	Good

*More specifically, noninvasive use in live subjects.

** Region of Interest

*** Limit of Detection

For *in vivo* measurements, both MRI and XRF provide satisfactory results when assessed relative to ferumoxytol dose or ICP-MS results. The imaging aspect of MRI allows for pinpointing of specific features, even if they are small and encapsulated by other regions or organs. XRF is limited to iron quantification in superficial or nearly superficial regions of an organism with lower overall specificity to the ROI and no image guidance. However, the output given by XRF may be preferred in that the reading will be a concentration of iron rather than an output like T2* time from MRI, which is not an exact concentration and is influenced by the oxidation state of iron and other tissue variations not related to iron concentration. Furthermore, extremely low concentrations of iron may not be detected by XRF, whereas extremely high concentrations of iron might exceed the upper limit of detection for MRI T2* map.

For biopsies, surgically resected tissue, or tissue culture samples, XRF, UV-Vis spectroscopy, and ICP-MS may be more appropriate. MRI could still be used in these cases, but it is not highly cost effective, and the imaging aspect is not typically needed as regions of interest can be easily isolated. XRF allows for the nondestructive analysis of these samples, allowing them to be retained for further analyses. However, if the equipment for XRF is not available and/or no further analyses will be conducted on the sample, UV-Vis spectroscopy is a cost-effective, relatively easy assay to perform. ICP-MS can also be used in place of XRF or UV-Vis as it is the gold standard and is widely used; however, it is destructive, significantly more expensive, and requires specialized equipment.

The use of a murine glioblastoma model in this study provides clear guidance for iron quantification by other preclinical researchers and lays the groundwork for future clinical investigation. Of the four tested methodologies, T2* from MRI is the most clinically relevant technique as large clinical datasets already describe the use of ferumoxytol as a qualitative MRI contrast agent and smaller MRI studies utilize it quantitatively [29,58–62]. The findings of the present study indicate that MRI and XRF are consistent and reliable methods for quantitative monitoring of ferumoxytol in mice. Translation of the tested approaches to human studies presents unique challenges including differences in iron metabolism, equipment variation and availability, larger tissue volumes, and differences in signal attenuation associated with increased superficial tissue and bone thickness. Notably, XRF has limited use in clinical research and would likely only be capable of superficial or potentially intraoperative use. However, XRF and ICP-MS may both be effective strategies for iron quantification in biopsies and other *ex vivo* samples in clinical settings. The novel side-by-side quantitative validation established by this study represents a starting point for critical future studies including further preclinical validation with larger sample sizes. Furthermore, it also provides informed guidance for selecting methodologies to further validate, standardize, and implement in clinical settings.

Altogether, these results provide definitive and proven methodologies for quantifying iron in a diverse array of sample types. Accessible, cost-effective options are provided in addition to the well-established, more expensive methods. Most importantly, a framework is provided for determining the optimal method to determine iron concentration in a diverse array of settings. This lends to the significance and impact of this work as these findings have implications in basic and preclinical research investigating the impacts of iron concentrations on diverse aspects of human health and disease. These findings may also provide guidance for the design of future clinical studies assessing these iron-related topics.

## Materials and methods

### Cell lines and culture conditions

GL-261 glioma cells were obtained from Creative Bioarray (Shirley, NY) and cultured in DMEM medium (CORNING, Product #10–013-CV) with 10% Fetal Bovine Serum (CORNING, Product #35–010-CV and 1% Penicillin/Streptomycin Solution (CORNING, Product #30–002-CI). RAW 264.7 macrophage cells were obtained from ATCC (Manassas, VA) and cultured under the same conditions.

### *In vivo* experiments

#### Tumor inoculation.

C57BL/6J mice were obtained from the Jackson Laboratory (Strain #000664). The animal use and care protocol was approved by the Purdue University Institutional Animal Care and Use Committee (Protocol Number: 2203002258). Thirty-eight mice between 12–20 weeks old were orthotopically injected with 5 x 10^5^ GL-261 cells. Briefly, GL-261 cells were trypsinized and suspended in PBS at a density of 1 x 10^8^ cells per mL. Mice were anesthetized with isoflurane and placed in a stereotactic frame with ear bars. A drill was used to create a burr hole through the skull located 2 mm to the right and 1 mm anterior of the bregma, and 5 µL of the cell suspension was injected by placing a syringe through the burr hole to a depth of 3.5 mm. The burr hole was closed with bone wax, and a topical tissue adhesive was used to close the surgical wound. Following tumor inoculation, mice received intraperitoneal injections of 10 mg/kg/day of meloxicam for 3 days as a post-operative analgesic. Mice were monitored at least 3 times per week by investigators and once per day by facility staff for the duration of the study (maximum of 140 days). Mice were euthanized immediately upon identification of predetermined humane endpoints associated with symptomatic disease, which included but were not limited to indications of pain, lethargy, and/or poor body condition score (< 2/5). Thirty-four mice were euthanized upon reaching humane endpoint criteria, and four mice were found dead. These outcomes are consistent with the rapid disease progression observed in this tumor model.

### Treatment and iron administration

All mice received treatment to mimic standard-of-care therapy for glioblastoma. Starting 6 days after tumor inoculation, mice underwent radiotherapy and chemotherapy treatment once per day on every weekday for the duration of 2 weeks. Six days post tumor inoculation was selected as the start date for therapy as MRI on day 6 confirmed a 100% tumor engraftment rate with a median tumor volume of 8 mm^3^. For radiotherapy treatment, mice were anesthetized with 90 mg/kg of ketamine (Covetrus, Catalog #071069; Dechra, Catalog #200−073) and 8 mg/kg of xylazine (Covetrus, Catalog #061035) via intraperitoneal injection. Full brain irradiation was performed with an XRad320 (Precision X-Ray, Madison, CT), using lead shielding to protect the remainder of the body. Mice received 10 x 2 Gy fractions (voltage = 320 kV, current = 12.5 mA, 2 mm aluminum filter) for a cumulative dose of 20 Gy. Temozolomide chemotherapy was delivered as an intraperitoneal injection at a dose of 50 mg/kg/day. The injection was prepared by diluting temozolomide powder (ThermoScientific, Catalog #466760050) in sterile saline to a final concentration of 5 mg/mL. Dissolution of the solution was achieved with 5 minutes of ultrasonification. The iron oxide nanoparticle ferumoxytol (Feraheme, AMAG Pharmaceuticals, Waltham, MA) was delivered as an intraperitoneal or intravenous injection once per day on every weekday for the duration of 4 weeks, the first 2 of which coincided with chemoradiotherapy treatment. Doses included a 0 mg/kg saline control and 20, 40, 60, 100, 200, and 300 mg/kg/day of ferumoxytol, with milligrams representing the amount of elemental iron. The ferumoxytol stock solution contained 30 mg of elemental iron per milliliter and was diluted with saline to appropriate injection volumes.

### MRI

MRI was performed at various timepoints during ferumoxytol administration. All MR images were obtained with a Bruker 7T Biospec 70/30 USR System (Ettlingen, Germany). Mice were anesthetized with 2–5% isoflurane gas, and T2 and T2* weighted images were acquired using manufacturer-provided TurboRARE and MGE scans, respectively. The TurboRare and MGE sequence parameters are provided in Table 2. T2* maps were calculated using the MGE scans with an in-house MATLAB code used for mono-exponential fitting on a voxel-by-voxel basis [27,63–65]. Tumor segments were manually delineated in Slicer on the T2-weighted images, and these segmentations were utilized on the co-registered T2* maps to calculate tumor values for T2* [66]. The median and minimum tumor T2* values were extracted from the segmentations for analysis.

**Table 2:** MRI parameters.

	TurboRARE	MGE
**Resolution**	0.0977 x 0.0977 x 0.5 mm^3^	0.1953 x 0.1953 x 0.5 mm^3^
**Field of view (FOV)**	25 x 25 mm^2^	25 x 25 mm^2^
**Number of slices**	25	25
**Slice thickness**	0.5 mm	0.5 mm
**Repetition time (TR)**	2750 ms	1500 ms
**Echo time (TE)**	11 ms	2.01 - 38.01 ms
**Number of echoes**	1	10 (4 ms intervals)
**Total acquisition time**	4.4 min	6.4 min

### Portable XRF

Five days after the last ferumoxytol treatment, mice were anesthetized with 90 mg/kg of ketamine and 8 mg/kg of xylazine. A ThermoScientific Niton XL3t XRF analyzer (Billerica, MA) was set to “Soils” detection mode, only operating on main (for iron detection), with a 30–60 second acquisition time. Mice were held in contact with the analyzer with the portion of the right hemisphere of their skull between the ear and the eye flush to the detector for the duration of the measurement. This positioning strategy minimized variation in beam placement and potential associated variation in measurement output.

### Preparation of iron phantoms

Ferumoxytol and low melting point agarose (Invitrogen, Catalog #15517−022) were added to water and heated in a microwave for dissolution of the agarose. Final concentrations for ferumoxytol were 0, 100, 250, 750, 1000, 1250, 2500, and 5000 ppm (assuming that 1 µg/mL is equal to 1 ppm, as ferumoxytol is an aqueous solution), and the final concentration of agarose was 4.2% weight per volume (w/v). After cooling and solidification of the agarose, a small cube of the gel for each ferumoxytol concentration was encased in a thin layer of water-resistant polyurethane and suspended in the center of a 1.5 mL microcentrifuge tube which was then filled with a solution with 0 ppm ferumoxytol and 4.2% w/v agarose. The cubes with standard concentrations of ferumoxytol were embedded in the gel containing 0 ppm of ferumoxytol for positive contrast of the MRI signal, and the polyurethane was used to prevent diffusion of the ferumoxytol.

### *Ex vivo* experiments

#### Brain extraction.

Mice were euthanized by carbon dioxide inhalation followed by cervical dislocation, and carcasses were stored at or below −20º C until all samples were ready to be processed. Brain tissue was excised, weighed, and stored at or below −20º C until analysis.

#### ICP-MS.

Brain tissue was submitted to the Research Instrumentation Center under the Purdue University Department of Chemistry (West Lafayette, IN) for ICP-MS analysis. Tissue samples were weighed and put into borosilicate digestion vessels (Anton Paar, Catalog #179436) and subsequently 0.5 mL of ultrapure water – provided by a Barnstead MircoPure water purification system (Thermo Scientific) – and 2 mL of ultra-high purity nitric acid (Aristar Ultra, VWR) was added to each sample. Method blanks containing only the high purity reagents were prepared similarly. Method blanks and samples were then digested simultaneously in an Anton Paar Multiwave 7000 microwave digestion system using the preconfigured ‘Organic’ program. After digestion, the samples and blanks were then diluted to 2.8% nitric acid by addition of ultrapure water to a volume of 50 mL. Internal standard (final concentration 5 ppb Indium) was added to samples and method blanks. These solutions were further diluted with ultrapure water (with 5ppb internal standard) to 2% nitric acid (and 5 ppb Indium) for analysis. Concentration calibrants with 0.01 ppb, 0.1 ppb, 1ppb, 10ppb, 100 ppb and 1 ppm Fe, were prepared by dilution with 2% nitric acid (with 5 ppb Indium added as internal standard) from an initial stock solution of 10 µg/mL Lutetium (in 5% nitric acid) purchased from Inorganic Ventures. ICP-MS analysis was conducted on a Thermo Scientific Element 2 mass spectrometer equipped with a Teledyne Cetac Aridus II nebulizer. Fe-56 and In-115 were measured in MID resolution mode using the default sampling times with ‘runs’ and ‘passes’ settings of 3 and 10, respectively. Take-up time was set to 90s. Linear calibration curves were constructed by normalizing calibrant intensities to the intensity of the internal standard and then subtracting the intensities measured in the blank (2.8% nitric acid for the calibrants, method blank for the samples). Intensities from samples were similarly normalized and method blank subtracted.

### UV-Visible spectroscopy

Immediately following euthanasia of a subset of the mice, brains were minced finely and placed in a 0.05% trypsin solution overnight at 4º C. The solution was then filtered through a 70 µm filter to isolate a single cell suspension and spun down to isolate the cell pellet. The protocol for iron quantification by UV-Vis spectroscopy was largely based on the published protocol by Torras et. al. [40]. Briefly, cell pellets were dissolved in concentrated HCl, and the solution was diluted with phosphate buffered saline (PBS) to obtain a 4.9 M HCl solution. Samples were spun down at 15,000 x g for 10 minutes to remove any remaining debris from the solution. Standard solutions of ferumoxytol ranging in concentration from 0–250 mg/L of iron were prepared using the same method. For data collection, 200 µL of the supernatant for each standard and sample were pipetted into a 96 well plate, and absorbance values at 348 nm were obtained with an Agilent Technologies Biotek Synergy Neo2 HTS plate reader (Santa Clara, CA) or a Molecular Devices SpectraMax ABS Plus plate reader (San Jose, CA). Final concentrations of iron were determined by the construction and implementation of a standard curve.

### *In vitro* experiments

#### Culture conditions for experiment.

Prior to starting the experiment, the cell culture conditions described above were followed. The protocol for this experiment is derived from a protocol by Feng et. al with several modifications [17]. In short, GL-261 and RAW 264.7 cells were seeded in T175 flasks at a density of 30,000 and 20,000 cells per cm^2^, respectively. Twenty-four hours later, 200 µg/mL of ferumoxytol was added. After an additional 48 hours, cells were trypsinized and counted. 8 million cells were set aside for UV-Vis spectroscopy and 50 million cells were set aside for benchtop XRF. Cell suspensions were spun down and the supernatant was removed to isolate the cell pellets.

### UV-Visible spectroscopy

Upon the isolation of the cell pellet, the same protocol described above for *ex-vivo* UV-Vis was utilized.

### Benchtop XRF

To closely replicate the protocol utilized for UV-Vis spectroscopy, samples were dissolved in concentrated HCl, and the solution was diluted with PBS to obtain a 4.9 M HCl solution. Samples were spun down at 15,000 x g for 10 minutes to remove any remaining debris from the solution. Standard solutions of ferumoxytol ranging in concentration from 0 to 2000 mg/L of iron were prepared using the same method. For data collection, 200 µL of the supernatant for each standard and sample were pipetted into XRF cups with a polypropylene film bottom, and the presence of iron was assessed with a Malvern Panayltical Epsilon 4 benchtop XRF (Almelo, The Netherlands). Final concentrations of iron were determined by the construction and implementation of standard curve using iron net signal.

### Manuscript preparation

#### Data analysis and figure generation.

R Studio and Slicer were used for data analysis, and R Studio, Slicer, Inkscape, Illustrae, and PowerPoint were utilized for figure generation [66–69]. Correlation assessments utilized the Spearman rank correlation due to a lack of normality in some of the data. For other analysis, the Student’s *t*-test was used on logarithmically transformed data unless otherwise specified.

The lower limit of detection (LOD) was estimated with Equation 1, where σ_residuals_ represents the standard deviation of the blank, and *m* represents the slope of the calibration curve.


$$\begin{array}{c} LOD=\text{3}\;\times \;\frac{\sigma_{residuals}}{m} \end{array}$$ (1)

### AI statement

Fig 3A was created using the generative AI tool Illustrae to convert a photo of the pXRF setup utilized in this study into a diagram [69].

## Supporting information

S1 Fig Preparation of calibration curve with T2* median from MRI using phantoms with known ferumoxytol concentrations. A) T2* median times for phantoms with up to 5000 ppm of ferumoxytol. B) T2* median times for phantoms up to 250 ppm of ferumoxytol. For both plots, lines indicate the best fit linear regression.
(TIF)

S2 Fig Representative slices of T2 anatomic MR images for each individual mouse at each timepoint receiving 0 mg/kg/day ferumoxytol. (TIF)

S3 Fig Representative slices of T2 anatomic MR images for each individual mouse at each timepoint receiving 20 mg/kg/day ferumoxytol. (TIF)

S4 Fig Representative slices of T2 anatomic MR images for each individual mouse at each timepoint receiving 40 mg/kg/day ferumoxytol. (TIF)

S5 Fig Representative slices of T2 anatomic MR images for each individual mouse at each timepoint receiving 60 mg/kg/day ferumoxytol. (TIF)

S6 Fig Representative slices of T2 anatomic MR images for each individual mouse at each timepoint receiving 100 mg/kg/day ferumoxytol. (TIF)

S7 Fig Representative slices of T2 anatomic MR images for representative mice receiving 200 (A) or 300 (B) mg/kg/day ferumoxytol. (TIF)

S8 Fig Analysis of changes in T2* values over time. T2* median values (A) and T2* minimum values (B) from day 6–34 post tumor inoculation. Data is reported as mean ± standard deviation. For all plots, N = 3–4 mice per treatment group.
(TIF)

S9 Fig Preparation of calibration curve with pXRF using phantoms with known ferumoxytol concentrations. (A) Measurements for phantoms up to 5000 ppm. (B) Measurements for phantoms up to 1250 ppm. For both plots, the lines indicate the best fit linear regression, and the shaded regions represent the 95% confidence interval for the regression.
(TIF)

S10 Fig Full spectra for UV-Vis spectrosocpy examination of ferumoxytol standards (A) and murine brain samples (B). The maximum absorbance peak of FeCl3 at 348 nm is indicated on both graphs. In panel B, individual lines represent brain samples from individual mice in that treatment group.
(TIF)

S1 File Supporting dataset for manuscript. Key and descriptions are included in the file.
(XLSX)

S2 File MRI processing pipeline documentation. MATLAB code utilized for MRI processing and analysis.
(DOCX)
