# Supplementary material for: Comparison of methods for in vivo and ex vivo quantification of iron in murine brain tumors
Source: PLoS One. 2026 Aug 17;21(8):e0356378. doi: 10.1371/journal.pone.0356378 (PMC13480596; doi:10.1371/journal.pone.0356378)
Supplement: Supplementary file 1 [file pone.0356378.s012.docx]

**MRI Processing Pipeline Documentation**

**Order of execution:**

readnmrpar →

nrrdWriterM →

fidreaderParavision6 →

t2_mems_cf_3F →

MGE_3F →

nrrdread →

extractingStats

**readnmrpar**

```
function P = readnmrpar(FileName)
% RBNMRPAR      Reads BRUKER parameter files to a struct
%
% SYNTAX        P = readnmrpar(FileName);
%
% IN            FileName:	Name of parameterfile, e.g., acqus
%
% OUT           Structure array with parameter/value-pairs
%

% Read file
A = textread(FileName,'%s','whitespace','\n');

% Det. the kind of entry
TypeOfRow = cell(length(A),2);

R = {   ...
    '^##\$*(.+)= \(\d\.\.\d+\)(.+)', 'ParVecVal' ; ...
    '^##\$*(.+)= \(\d\.\.\d+\)$'   , 'ParVec'    ; ...
    '^##\$*(.+)=(.+)'             , 'ParVal'    ; ...
    '^([^\$#].*)'                   , 'Val'       ; ...
    '^\$\$(.*)'                     , 'Stamp'     ; ...
    '^##\$*(.+)='                   , 'EmptyPar'  ; ...
	'^(.+)'							, 'Anything'	...
    };

for i = 1:length(A)
    for j=1:size(R,1)
        [s,t]=regexp(A{i},R{j,1},'start','tokens');
        if (~isempty(s))
            TypeOfRow{i,1}=R{j,2};
            TypeOfRow{i,2}=t{1};
        break;
        end
    end
end

% Set up the struct
i=0;
while i < length(TypeOfRow)
    i=i+1;
    switch TypeOfRow{i,1}
        case 'ParVal'
            LastParameterName = TypeOfRow{i,2}{1};
            P.(LastParameterName)=TypeOfRow{i,2}{2};
        case {'ParVec','EmptyPar'}
            LastParameterName = TypeOfRow{i,2}{1};
            P.(LastParameterName)=[];
        case 'ParVecVal'
            LastParameterName = TypeOfRow{i,2}{1};
            P.(LastParameterName)=TypeOfRow{i,2}{2};
        case 'Stamp'
            if ~isfield(P,'Stamp')
                P.Stamp=TypeOfRow{i,2}{1};
            else
                P.Stamp=[P.Stamp ' ## ' TypeOfRow{i,2}{1}];
            end
        case 'Val'
			if isempty(P.(LastParameterName))
				P.(LastParameterName) = TypeOfRow{i,2}{1};
			else
				P.(LastParameterName) = [P.(LastParameterName),' ',TypeOfRow{i,2}{1}];
			end
        case {'Empty','Anything'}
            % Do nothing
    end
end


% Convert strings to values
Fields = fieldnames(P);

for i=1:length(Fields);
    trystring = sprintf('P.%s = [%s];',Fields{i},P.(Fields{i}));
    try
        eval(trystring);
	catch %#ok<CTCH>
        % Let the string P.(Fields{i}) be unaltered
    end
end
```

**nrrdWriterM**

```
% ========================================================================
%
% nrrdwriter_dan
%
% filename  - 'myimage.ext' - 'veins.nrrd'
% matrix    - data - Matlab matrix
% pixelspacing - boxel size
% origin    - point from the image is generated
% encoding  - raw, ascii, gzip
%
% ========================================================================

function ok = nrrdWriterM(filename, matrix, pixelspacing, origin, encoding)

% This line gets the path, name and extension of our file:
% pathf = /home/mario/.../myfile.myext
% fname = myfile
% ext = .myext
[pathf, fname, ext] = fileparts(filename);

format=ext(2:end); % We remove the . from .ext
% so we extract the output format from the argument filename, instead of
% put two different arguments

matrix = permute(matrix, [2 1 3]); % so we undo permute of index in nrrdreader;

dims=(size(matrix)); % matrix dimensions (size NxMxP)
ndims=length(dims);     % number of dimensions (dim n)


% =====================================================================
% Conditions to make sure our file is goint to be created succesfully.
%
% First the code puts the argument 'encoding' in lowercase
encoding = lower(encoding);

encodingCond = isequal(encoding, 'ascii') || isequal(encoding, 'raw') || isequal(encoding, 'gzip');
assert(encodingCond, 'Unsupported encoding')

% The same with output format
format = lower(format);
formatCond = isequal(format,'nhdr') || isequal(format,'nrrd');
assert(formatCond, 'Unexpected format');

% ======================================================================

% Now, if our conditions are satisfied:
if (encodingCond && formatCond)

    % Header

    % Open, filename (which specifies output format) and write binary
    fid = fopen(filename, 'wb','l');
    fprintf(fid,'NRRD0004\n');      % NRRD type 4

    % Type of variable we're storing in our file
    mtype=class(matrix);
    outtype=setDatatype(mtype);
    fprintf(fid,['type: ', outtype, '\n']);

    %
    fprintf(fid,['dimension: ', num2str(ndims), '\n']);

    if isequal(ndims, 2)
        fprintf(fid,'space: left-posterior\n');
    elseif isequal (ndims, 3)
        fprintf(fid,'space: left-posterior-superior\n');
    end

    fprintf(fid,['sizes: ',num2str(dims), '\n']);

    if isequal(ndims, 2)
        fprintf(fid,['space directions: (',num2str(pixelspacing(1)), ...
            ',0) (0,',num2str(pixelspacing(2)), ')\n']);
        fprintf(fid,'kinds: domain domain\n');
    elseif isequal (ndims, 3)
        fprintf(fid,['space directions: (',num2str(pixelspacing(1)), ...
            ',0,0) (0,',num2str(pixelspacing(2)), ',0) (0,0,',...
            num2str(pixelspacing(3)), ')\n']);
        fprintf(fid,'kinds: domain domain domain\n');
    end

    fprintf(fid,['encoding: ', encoding, '\n']);

    [~,~,endian] = computer();

    if (isequal(endian, 'B'))
        fprintf(fid,'endian: big\n');
    else
        fprintf(fid,'endian: little\n');
    end

    if isequal(ndims, 2)
        fprintf(fid,['space origin: (', num2str(origin(1)),',', num2str(origin(2)),')\n']);
    elseif isequal (ndims, 3)
        fprintf(fid,['space origin: (', num2str(origin(1)), ...
            ',',num2str(origin(2)),',', num2str(origin(3)),')\n']);
    end

    if (isequal(format, 'nhdr')) % Si hay que separar
        % Escribir el nombre del fichero con los datos
        fprintf(fid, ['data file: ', [fname, '.', encoding], '\n']);

        fclose(fid);
        if isequal(length(pathf),0)
            fid = fopen([fname, '.', encoding], 'wb');
        else
            fid = fopen([pathf, filesep, fname, '.', encoding], 'wb');
        end
    else
        fprintf(fid,'\n');
    end

    ok = writeData(fid, matrix, outtype, encoding);
    fclose(fid);
end


% ========================================================================
% Determine the datatype --> From mtype (matlab) to outtype (NRRD) -->
% ========================================================================
function datatype = setDatatype(metaType)

% Determine the datatype
switch (metaType)
%  case {'int8', 'uint8', 'int16', 'uint16', 'int32', 'uint32', 'int64',...
%        'uint64', 'double'}
%    datatype = metaType;

 case {'uint8', 'int16', 'uint16', 'int32', 'uint32', 'int64',...
       'uint64', 'double'}
   datatype = metaType;

  case {'int8'}
  datatype = 'short';


 case {'single'}
  datatype = 'float';

 otherwise
  assert(false, 'Unknown datatype')
end

%
% ========================================================================
% writeData -->
% fidIn is the open file we're overwriting
% matrix - data that have to be written
% datatype - type of data: int8, string, double...
% encoding - raw, gzip, ascii
% ========================================================================
function ok = writeData(fidIn, matrix, datatype, encoding)

switch (encoding)
 case {'raw'}

  ok = fwrite(fidIn, matrix(:), datatype);

 case {'gzip'}
     % Store in a raw file before compressing
     %tmpBase = tempname(QtbiServices.get_working_directory);
     tmpBase = tempname;
     tmpFile = [tmpBase '.gz'];

     [fidTmpRaw, msg] = fopen(tmpBase, 'wb');

     assert(fidTmpRaw > 3, 'Could not open temporary file for GZIP compression');

     %fwrite(fidTmpRaw, matrix(:), datatype);
     fwrite(fidTmpRaw, matrix(:), datatype);
     fclose(fidTmpRaw);

     % Now we gzip our raw file
     gzip(tmpBase);

     % Finally, we put this info into our nrrd file (fidIn)
     fidTmpRaw = fopen(tmpFile, 'rb');
     %tmp = fread(fidTmpRaw, Inf, [datatype '=>' datatype]);
     tmp = fread(fidTmpRaw, Inf, 'uint8=>uint8');
     %ok = fwrite (fidIn, tmp, datatype);

     ok = fwrite (fidIn, tmp, 'uint8');
     %cleaner = onCleanup(@() fclose(fidTmpRaw));
     fclose(fidTmpRaw);
     delete (tmpBase);
     delete(tmpFile);

     %catch ME
     %    msgbox(ME.msgtext);
     %end
 case {'ascii'}

  ok = fprintf(fidIn,'%u ',matrix(:));
  %ok = fprintf(fidIn,matrix(:), class(matrix));

 otherwise
  assert(false, 'Unsupported encoding')
end
```

**fidreaderParavision6**

```
function [] = fidreaderParavision6
%%%%%%%%%%%%%%%%Generates Signals and Parameters files for all relevent scans
%%%%%%%%%%%%%%AF Q1 2018 Adaptation from BrukerImageProcessor AM Stokes
%%%%%%%%%%%%Make sure fid is 16 bit otherwise manually read the file as
%%%%%%%%%%%%32bit with fread function

%Get the current directory (Mouse/Patient File)
dirname = 'C:\Path\To\Your\Project\';

%%% Search through directory for scans
for a = 1:40
    try
        BRUKER_FILE1=[dirname '/' num2str(a) '/pdata/1/2dseq'];   %This program uses the processed 2dseq file.
        BRUKER_FILE1=[dirname '\' num2str(a) '\pdata\1\2dseq'];   %This program uses the processed 2dseq file.
        pars = readnmrpar([BRUKER_FILE1(1:end-13) 'method']);
        display(['The method for scan number ' num2str(a) ' is ' pars.Method '. and time is ',pars.PVM_ScanTimeStr]);
    catch
    end
end
%%%%%Choose the scans you want processed
scans = input('Enter the Scan number you would like processed in a vector ex : [ 1 ...n]');
%%%Processing For loop
for k = 1: length(scans)
    clear BRUKER_FILE1 pars Nechoes
    BRUKER_FILE1=[dirname '/' num2str(scans(k)) '/pdata/1/2dseq'];
    pars = readnmrpar([BRUKER_FILE1(1:end-13) 'method']);
    %visu = readnmrpar([BRUKER_FILE1(1:end-5) 'visu_pars']);
    NpointRD=pars.PVM_Matrix(2);       %Size of read direction matrix
    NpointPE=pars.PVM_Matrix(3);      %Number of phase encode steps
    ns = pars.PVM_SPackArrNSlices(2); %Slices
    if length(pars.PVM_Matrix) > 3 %%for 3D acq
        ns=pars.PVM_Matrix(4);
    end

%%% Account for the Possible Inputs
switch pars.Method
    case 'gESFIDE'
        Nechoes = pars.NEchoes;
        dyn = pars.PVM_NRepetitions;
        voxel_size = [pars.PVM_SpatResol(2:3) pars.PVM_SliceThick]
    case '<Bruker:MGE>'
        Nechoes = pars.PVM_NEchoImages;
        dyn = pars.PVM_NRepetitions;
        voxel_size = [pars.PVM_SpatResol(2:3) pars.PVM_SliceThick]
    case 'MSME'
        Nechoes = pars.NEchoes;
        dyn = pars.PVM_NRepetitions;
    case '<Bruker:RAREVTR>'
        Nechoes = pars.MultiRepTime(1);
        dyn = pars.PVM_NRepetitions;
        pars.RECO_wordtype(2:3) = '16';
        voxel_size = [pars.PVM_SpatResol(2:3) pars.PVM_SliceThick]
    case '<Bruker:RARE>'
        dyn = pars.PVM_NRepetitions;
        Nechoes = pars.PVM_NEchoImages;
        TE = pars.EffectiveTE
        TR = pars.PVM_RepetitionTime
        voxel_size = [pars.PVM_SpatResol(2:3) pars.PVM_SliceThick]
    case '<Bruker:EPI>'
        dyn = 0;
        Nechoes = pars.PVM_NRepetitions;
        voxel_size = [pars.PVM_SpatResol(2:3) pars.PVM_SliceThick]
    case 'DtiStandard'
        dyn = 0;
        ns = pars.PVM_SPackArrNSlices(2); %Slices

        Nechoes = pars.PVM_DwNDiffExp;
        NpointRD=visu.VisuCoreSize(2);       %Size of read direction matrix
        NpointPE=visu.VisuCoreSize(3); % Size of Phase Direction
    case 'DtiEpi'
        dyn = 0;
        ns = pars.PVM_SPackArrNSlices(2); %Slices
        Nechoes = length(pars.PVM_DwEffBval)-1;
        NpointRD = pars.PVM_Matrix(3);
        NpointPE = pars.PVM_Matrix(2);
     case '<Bruker:FLASH>'
        dyn = pars.PVM_NRepetitions;
        Nechoes = pars.PVM_NEchoImages;
        TE = pars.PVM_EchoTime
        TR = pars.PVM_RepetitionTime
        try
            voxel_size = pars.PVM_SpatResol(2:4)
        catch
            voxel_size = [pars.PVM_SpatResol(2:3) pars.PVM_SliceThick]
        end
     case 'FieldMap'
        dyn = 0;
        Nechoes = pars.PVM_NEchoImages;
        voxel_size = pars.PVM_SpatResol(2:4)


end
%%%%Assigning bit integer
try
    pars.RECO_wordtype;
catch
    pars.RECO_wordtype(2:3) = '16';
end
%%%%%%Read Fid
fid1=fopen(BRUKER_FILE1,'r','l');   %Open fid
fidmix1=fread(fid1,inf,['int' pars.RECO_wordtype(2:3)]);    %Read fid data as single line
if dyn>2 && ns > 1
    signal=reshape(fidmix1,NpointRD,NpointPE,Nechoes,ns,dyn);    %Reshape data into read and phase encode directions
else
    if strcmp(pars.Method, '<Bruker:RAREVTR>') || strcmp(pars.Method, '<Bruker:EPI>')
        signal=reshape(fidmix1,NpointRD,NpointPE,ns,Nechoes);        %Reshape data into read and phase encode directions
    else
        signal=reshape(fidmix1,NpointRD,NpointPE,Nechoes,ns);
    end
end

signal = squeeze(signal);
matrix_size = size(signal)
% averages = pars.PVM_NAverages

if Nechoes > 1 && Nechoes < 20 %if there is more than one echo save nrrd files for each echo
    for j = 1:Nechoes
        disp(['now starting echo ', num2str(j)])
        if strcmp(pars.Method, '<Bruker:RAREVTR>')
            signalEcho = signal(:,:,:,(Nechoes-j+1));
        else
            signalEcho = signal(:,:,j,:);
        end

        signalEcho = squeeze(signalEcho);
        matrix_size = size(signalEcho)
%         averages = pars.PVM_NAverages

        signalEcho = imrotate3(signalEcho,270,[0,0,1],'nearest', 'loose');  %Rotate image 270 degrees to match pet
%         corrected = -1*ones(size(signalEcho));
%         for i=1:matrix_size(3)
%             corrected(:,:,i) = signalEcho(:,:,(matrix_size(3)-i+1));  % use flip function instead !!!!!!!!
%         end
%         signalEcho = corrected;
        nrrdWriterM([dirname, '/' num2str(scans(k)) '\signalEcho',num2str(j),'.nrrd'],signalEcho,voxel_size,[0 0 0], 'gzip');
    end
end

% for large dynamic datasets save just middle slice with tumor so  it is 3D
% if dyn > 2
%     tumor_slice = 2; % best slice with tumor
%     signal = squeeze(signal(:,:,tumor_slice,:));
% end

% for large dynamic datasets save just sing time frame so  it is 3D
if dyn > 2
    time_slice = 1; %
    signal = squeeze(signal(:,:,:,time_slice));
end

if length(size(signal)) <= 3
    % geometric changes
    signal = imrotate3(signal,270,[0,0,1],'nearest', 'loose');  %Rotate image 270 degrees to match pet
    % switch slice order to match what you want
%     corrected = -1*ones(size(signal));
%     for i=1:size(signal,3)
%         corrected(:,:,i) = signal(:,:,(size(signal,3)-i+1));  % use flip function instead !!!!!!!!
%     end
%     signal = corrected;

    % for ex vivo in brain slicer block may need rotate again
%     signal = imrotate3(signal,180,[0,0,1],'nearest', 'loose');  %Rotate image 180 degrees to match pet

    nrrdWriterM([dirname '/' num2str(scans(k)) '/signal.nrrd'],signal,voxel_size,[0 0 0], 'gzip');
end


save([dirname '/' num2str(scans(k)) '\signal.mat'],'signal','pars')


end
    clear k
end
```

**t2_mems_cf_3F**

```
function M = t2_mems_cf_3F(params,TE)
t = TE;
T2 = params(1);
So = params(2);
A = params(3);
M = So*exp(-t/T2)+A;

% % figure(1); plot(TE,s,'x',TE,M,'LineWidth',2);
% %                 legend('Data','Model');
% %                 title(['T2 = ' num2str(T2)]);
% %                 drawnow

end
```

**MGE_3F**

```
function [] = MGE_3F
%%%%%%%%%%%%%Multi Gradient Echo Fitting%%%%%%%%%%%%%%%%%%
%%%%%%%%%%%%%AF / AS Q1 2018%%%%%%%%%%%%%%%%
% Take in signal parameters to fit data to find T2s  you can choose the
% number of echoes you fit using ef
% updated JPS 2023

close all
clear all
dir = 'C:\Path\To\Your\Project\';

signal =[];
load([dir,'signal.mat']);

%Prep
voxel_size = [pars.PVM_SpatResol(2:3) pars.PVM_SliceThick];
TEeff = pars.EffectiveTE(2:end); %Effective Echo Times
options = optimset('TolFun',1e-9,'Tolx',1e-9,'MaxIter',1000,'Display','off');
[nx, ny, ne, nz ]  = size(signal);
A = 0;
T2s = zeros(nx,ny,nz);
S0 = zeros(nx,ny,nz);
A0 = zeros(nx,ny,nz);
R0 = zeros(nx,ny,nz);
RSS = 0;
    parfor z = 1:nz % -3
        disp(z)
        for x = 1:nx  % 20:85; 1:nx
            %disp(x);
            for y = 1:ny % 10:60 1:ny
                data = squeeze(signal(x,y,:,z));
                if isnan(data(1))

                else
                    voxel_data = data';
                    params = [12 max(voxel_data) A]; %initial guesses [T2s So DC noise component]
                    LB = [0 0 0];
                    UB = [Inf Inf Inf];
                    [output, resnorm] = lsqcurvefit('t2_mems_cf_3F',params,TEeff,voxel_data,LB,UB,options);
                    T2s(x,y,z) = output(1);
                    S0(x,y,z) = output(2);
                    A0(x,y,z) = output(3);
                    R0(x,y,z) = resnorm;
                    RSS = RSS+resnorm;

                end
            end
        end
    end


% save([saveDir,'MGET2s.mat'],'T2s','S0','TEeff')

% write to nrrd file

% geometry manipulations (optional)
T2s = imrotate3(T2s,270,[0,0,1],'nearest', 'loose');  % Rotate image 270 degrees to match pet
% switch slice order to match what you want
% corrected = -1*ones(size(T2s));
% for i=1:nz
%     corrected(:,:,i) = T2s(:,:,nz-i+1);  % use flip function instead !!!!!!!!
% end
% T2s = corrected;


if size(T2s,3) == 1
    temp = zeros([size(T2s),2]);
    temp(:,:,1)=T2s;
    T2s = temp;
end
nrrdWriterM([dir, 'T2map3F.nrrd'],T2s, voxel_size, [0 0 0], 'gzip');
nrrdWriterM([dir, 'Bkgd.nrrd'],A0, voxel_size, [0 0 0], 'gzip');
nrrdWriterM([dir, 'Residualmap3F.nrrd'],R0, voxel_size, [0 0 0], 'gzip');
display(['the sum of residuals is ', num2str(RSS)])
end
```

**nrrdread**

```
function [X, meta] = nrrdread(filename)
%NRRDREAD  Import NRRD imagery and metadata.
%   [X, META] = NRRDREAD(FILENAME) reads the image volume and associated
%   metadata from the NRRD-format file specified by FILENAME.
%
%   Current limitations/caveats:
%   * "Block" datatype is not supported.
%   * Only tested with "gzip" and "raw" file encodings.
%   * Very limited testing on actual files.
%   * I only spent a couple minutes reading the NRRD spec.
%
%   See the format specification online:
%   http://teem.sourceforge.net/nrrd/format.html


% Open file.
fid = fopen(filename, 'rb');
assert(fid > 0, 'Could not open file.');
cleaner = onCleanup(@() fclose(fid));

% Magic line.
theLine = fgetl(fid);
assert(numel(theLine) >= 4, 'Bad signature in file.')
assert(isequal(theLine(1:4), 'NRRD'), 'Bad signature in file.')

% The general format of a NRRD file (with attached header) is:
%
%     NRRD000X
%     <field>: <desc>
%     <field>: <desc>
%     # <comment>
%     ...
%     <field>: <desc>
%     <key>:=<value>
%     <key>:=<value>
%     <key>:=<value>
%     # <comment>
%
%     <data><data><data><data><data><data>...

meta = struct([]);

% Parse the file a line at a time.
while (true)

    theLine = fgetl(fid);

    if (isempty(theLine) || feof(fid))
        % End of the header.
        break;
    end

    if (isequal(theLine(1), '#'))
        % Comment line.
        continue;
    end

    % "fieldname:= value" or "fieldname: value" or "fieldname:value"
    parsedLine = regexp(theLine, ':=?\s*', 'split','once');

    assert(numel(parsedLine) == 2, 'Parsing error')

    field = lower(parsedLine{1});
    value = parsedLine{2};

    field(isspace(field)) = '';
    meta(1).(field) = value;

end

datatype = getDatatype(meta.type);

% Get the size of the data.
assert(isfield(meta, 'sizes') && ...
    isfield(meta, 'dimension') && ...
    isfield(meta, 'encoding') && ...
    isfield(meta, 'endian'), ...
    'Missing required metadata fields.')

dims = sscanf(meta.sizes, '%d');
ndims = sscanf(meta.dimension, '%d');
assert(numel(dims) == ndims);

data = readData(fid, meta, datatype);
data = adjustEndian(data, meta);

% Reshape and get into MATLAB's order.
X = reshape(data, dims');
X = permute(X, [2 1 3]); %%popravila in pustila tako kot je


function datatype = getDatatype(metaType)

% Determine the datatype
switch (metaType)
    case {'signed char', 'int8', 'int8_t'}
        datatype = 'int8';

    case {'uchar', 'unsigned char', 'uint8', 'uint8_t'}
        datatype = 'uint8';

    case {'short', 'short int', 'signed short', 'signed short int', ...
            'int16', 'int16_t'}
        datatype = 'int16';

    case {'ushort', 'unsigned short', 'unsigned short int', 'uint16', ...
            'uint16_t'}
        datatype = 'uint16';

    case {'int', 'signed int', 'int32', 'int32_t'}
        datatype = 'int32';

    case {'uint', 'unsigned int', 'uint32', 'uint32_t'}
        datatype = 'uint32';

    case {'longlong', 'long long', 'long long int', 'signed long long', ...
            'signed long long int', 'int64', 'int64_t'}
        datatype = 'int64';

    case {'ulonglong', 'unsigned long long', 'unsigned long long int', ...
            'uint64', 'uint64_t'}
        datatype = 'uint64';

    case {'float'}
        datatype = 'single';

    case {'double'}
        datatype = 'double';

    otherwise
        assert(false, 'Unknown datatype')
end


function data = readData(fidIn, meta, datatype)

switch (meta.encoding)
    case {'raw'}

        data = fread(fidIn, inf, [datatype '=>' datatype]);

    case {'gzip', 'gz'}

        tmpBase = tempname();
        tmpFile = [tmpBase '.gz'];
        fidTmp = fopen(tmpFile, 'wb');
        assert(fidTmp > 3, 'Could not open temporary file for GZIP decompression')

        tmp = fread(fidIn, inf, 'uint8=>uint8');
        fwrite(fidTmp, tmp, 'uint8');
        fclose(fidTmp);

        gunzip(tmpFile)

        fidTmp = fopen(tmpBase, 'rb');
        %cleaner = onCleanup(@()fclose(fidTmp));

        meta.encoding = 'raw';
        data = readData(fidTmp, meta, datatype);

        fclose(fidTmp);
        system(['del ' tmpFile]);
        system(['del ' tmpBase]);

    case {'txt', 'text', 'ascii'}

        data = fscanf(fidIn, '%f');
        data = cast(data, datatype);

    otherwise
        assert(false, 'Unsupported encoding')
end


function data = adjustEndian(data, meta)

[~,~,endian] = computer();

needToSwap = (isequal(endian, 'B') && isequal(lower(meta.endian), 'little')) || ...
    (isequal(endian, 'L') && isequal(lower(meta.endian), 'big'));

if (needToSwap)
    data = swapbytes(data);
end
```

**extractingStats**

```
% Modified by MLS 5/27/19
% Updated for preclinical fluciclovine study
%
% Modified MLS 1/6/23 for Purdue
%
%
% Inputs: 1) image file, 2) segmentation/label field file
% Ouputs: Writes statistics to excel file
%
%

clear
%%	Reading in data (directories and names of files)
% Generic study
study = 'GenericStudy';
baseDir='C:\Path\To\Your\Project\'; % where the mri files are at
metrics2Extract = {'max';'min'; 'median'; 'total'; 'volume'; 'mean';'stdev';'adjmean';'adjmedian';'adjmax';'totalpercent'};
patients = {'Patient1';'Patient2';'Patient3';'Patient4';'Patient5';'Patient6';'Patient7' }; %  name of patient/subject for importing to MATLAB
outPatient = {'Patient1';'Patient2';'Patient3';'Patient4';'Patient5';'Patient6';'Patient7'}; % name of patient/subject when writing to output excel file
interestRegion = 'tumor';  %   will be placed in ouput excel file for reference
saveDir = [baseDir, 'MRI results.xlsx']; % name/dir of excel file to save output excel file
scans = {'20241212' }; % if multiple imaging timepoints
threshold = 1000; % threshold for cutting out values greater than

%% start code
writeDex = 2;
for patient_ind = 1:size(patients,1)
    for scan_ind = 1: length(scans)
        patient = cell2mat(patients(patient_ind,:));
        scan = cell2mat(scans(scan_ind));
        disp(['Now starting ',patient, '_', scan]);

%% read in ROI and check if it exists
switch study
            case('GenericStudy')
                segmentation_directory = [baseDir, patient, '_', scan,'_seglabel.nrrd'];    % directory of ROI
                img = [baseDir, patient, '_', scan, '_T2map.nrrd'];   % directory of image
        end

        if exist(segmentation_directory, 'file') ~= 0

            % read in ROI and image
            [ROI, meta] = nrrdread([segmentation_directory]);
            a = meta.spacedirections;
            p = [",","(",")"];
            b = str2num(replace(a,p," "));
            voxel_sizes = [b(1),b(5),b(9)];
            [imgData, ~] = nrrdread(img);


            %Determine the number of segmentations in the labelfield
            ind = find(ROI);
            m = unique(ROI);  % m = number of segs
            m=rot90(m);

            for i = 2:(length(m)) %
                tumor = m(i);

                % Determine the indices of the image voxels corresponding to the ROI/tumor
                ind = find(ROI==tumor);

                v = voxel_sizes;
                voxel_volume = v(1)*v(2)*v(3);
                ROIdata = imgData(ind);

                % Find the max, mean, and total in the tumor and volume of tumor

                metrics = cell(size(metrics2Extract));
                metricVals = zeros(size(metrics2Extract));
                for  j = 1:length(metrics2Extract)
                    if strcmp(metrics2Extract(j), 'max')
                        SUVmax = max(imgData(ind));
                        metrics(j)  =   metrics2Extract(j);
                        metricVals(j) =  SUVmax;
                    elseif strcmp(metrics2Extract(j), 'min')
                        SUVmin = min(imgData(ind));
                        metrics(j)  =   metrics2Extract(j);
                        metricVals(j) =  SUVmin;
                    elseif strcmp(metrics2Extract(j), 'median')
                        SUVmedian = median(imgData(ind));
                        metrics(j)  =  metrics2Extract(j);
                        metricVals(j) = SUVmedian;
                    elseif strcmp(metrics2Extract(j), 'total')
                        SUVtot = sum(imgData(ind))*voxel_volume;
                        metrics(j)  =  metrics2Extract(j);
                        metricVals(j) = SUVtot;
                    elseif strcmp(metrics2Extract(j), 'volume')
                        volume = size(ind,1)* size(ind,2)*voxel_volume;
                        metrics(j)  =   metrics2Extract(j);
                        metricVals(j) = volume;
                     elseif strcmp(metrics2Extract(j), 'qqd')
                        qqd = (quantile(imgData(ind), 0.75) - quantile(imgData(ind), 0.25)) /(quantile(imgData(ind), 0.75) +quantile(imgData(ind), 0.25) ) ;
                        metrics(j)  =  metrics2Extract(j);
                        metricVals(j) = qqd;
                    elseif strcmp(metrics2Extract(j), 'mean')
                        me = mean(imgData(ind));
                        metrics(j) = metrics2Extract(j);
                        metricVals(j) = me;
                    elseif strcmp(metrics2Extract(j), 'stdev')
                        stdev = std(imgData(ind));
                        metrics(j) = metrics2Extract(j);
                        metricVals(j) = stdev;
                    elseif strcmp(metrics2Extract(j), 'adjmean')
                        temp = imgData(ind);
                        meadj = mean(temp(temp<threshold));
                        metrics(j) = metrics2Extract(j);
                        metricVals(j) = meadj;
                    elseif strcmp(metrics2Extract(j), 'adjmedian')
                        temp = imgData(ind);
                        SUVmedianadj = median(temp(temp<threshold));
                        metrics(j) = metrics2Extract(j);
                        metricVals(j) = SUVmedianadj;
                    elseif strcmp(metrics2Extract(j), 'adjmax')
                        temp = imgData(ind);
                        SUVmaxadj = max(temp(temp<threshold));
                        metrics(j) = metrics2Extract(j);
                        metricVals(j) = SUVmaxadj;
                    elseif strcmp(metrics2Extract(j), 'totalpercent') %the percent of voxels that are crashed out
                        temp = imgData(ind);
                        SUVtotpercent = 100*(numel(imgData(ind))-numel(temp(temp<threshold)))/numel(imgData(ind));
                        metrics(j)  =  metrics2Extract(j);
                        metricVals(j) = SUVtotpercent;
                    end
                end

                info = {cell2mat(outPatient(patient_ind,:)), scan, interestRegion,i, ' '};
                info = repmat(info,length(metrics2Extract),1);
                info(:,size(info,2)) = metrics;
                info =  table(info);
                metricVals = table(metricVals);

                writetable(info, saveDir, 'Sheet', interestRegion, 'Range', ['A',num2str(writeDex),':F', num2str(writeDex+length(metrics2Extract))], 'writeVariableNames',false);
                writetable(metricVals, saveDir, 'Sheet', interestRegion, 'Range', ['G',num2str(writeDex),':G',num2str(writeDex+length(metrics2Extract))], 'writeVariableNames',false);
                writeDex = writeDex + length(metrics2Extract);

            end

        else
            disp(['no segmentation found for', patients(patient_ind,:), scans(scan_ind)]);
        end
    end
end
```
