# Supplementary material for: Comparison of methods for in vivo and ex vivo quantification of iron in murine brain tumors
Source: PLoS One. 2026 Aug 17;21(8):e0356378. doi: 10.1371/journal.pone.0356378 (PMC13480596; doi:10.1371/journal.pone.0356378)
Supplement: Supplementary file 2 [file pone.0356378.s014.docx]

**Response to comments**

The authors greatly appreciate the time and effort of the editor and the reviewers to assess our manuscript and give valuable feedback. We have taken each comment into careful consideration and modified the manuscript accordingly. Please see responses to individual comments below.

**Reviewer 1 comments**

1. **After reading the introduction, the rationale for administering ferumoxytol in the different models remains unclear.**

We agree that this rationale was underdeveloped in the manuscript. We have addressed this by expanding on the theragnostic potential of ferumoxytol and discussing the importance of quantification for monitoring disease progression and therapeutic concentrations (please see the comments in the revised manuscript, which indicate the specific sections that were added/altered to address each comment).

2. **The authors mention that this drug is administered as an MRI contrast agent. What is the incidence of this drug's use as a contrast agent in clinical practice?**

Although the total incidence of ferumoxytol’s off-label use as an MRI contrast agent is difficult to determine, we have now included manufacturer-reported incidence values for all ferumoxytol use (2.6 million uses), as well as incidence of ferumoxytol use as an MRI contrast agent in a multicenter safety analysis (4000+ uses over a 15-year period across six centers).

3. **What is the significance of detecting elevated iron levels induced by ferumoxytol when iron levels in the brain are actually very low? If I understand correctly, the nanoparticle is primarily a tool for validating and standardizing iron quantification. If so, please specify this clearly in the text.**

We added a sentence to the Introduction about our model being an ideal system for developing, testing, and comparing our methods for iron quantification. We believe our additions to address comment #1 also help to address this comment. Ferumoxytol is being tested as a therapeutic agent, so the methods developed here are geared towards assessing iron in those applications or diseases where iron concentrations might be higher than normal brain levels e.g. brain tumor.

4. **The dosing scheme (up to 300 mg/kg/day × 21 injections) is extremely high relative to clinical use of ferumoxytol. What is the biological relevance to human imaging or iron physiology? Therefore, it is recommended to clearly justify the selection of doses and, based on that, discuss the clinical translation of the results.**

We agree, these doses are relatively high compared to current human usage. This is for multiple reasons, including 1) our use of ferumoxytol as a cancer therapeutic, which requires higher doses than current applications and 2) the differences in body surface area between mice and humans. We have included a brief mention of the dose range when converted to human equivalent dose based on organism surface area to help in part account for faster metabolism of the drug in mice (using guidance from the FDA). This is partly related to the short plasma half-life of ferumoxytol in mice compared to humans, which has also been noted in the revised manuscript. Once converted to the human equivalent dose, the tested dose ranges in this study become more clinically relevant.

5. **The authors mention that a descending trend was also observed in mice receiving 0 mg/kg ferumoxytol. This appears to be critical because it suggests that changes in T2 are not specific to iron accumulation. What factors in tumor progression might be influencing this signal? Histological validation would be valuable to elucidate this result.**

We have clarified some potential non-iron factors that could contribute to decreasing T2* in the control mice in the Discussion and have included references for other studies, which document these effects. Unfortunately, histological validation was not possible as samples were destroyed for further iron analyses; however, some of the aforementioned references do include pathologic correlations with T2*.

6. **What is the probability that in vivo analysis with the Portable XRF is dependent on the position of the beam and the rodent's head?**

This is an important point, since the position of the beam on the rodent’s head will affect the Portable XRF output. To mitigate positional uncertainties, we developed a strategy to ensure reproducible placement of the Portable XRF. We have emphasized in the Methods section that we used this positioning strategy to minimize variation in beam placement and therefore any associated variation in measurement output. We have also added this as a potential source of uncertainty to the Portable XRF measurements in the Discussion section of the revised manuscript.

7. **Is it likely that iron will accumulate in the hair and scalp after ferumoxytol administration? If so, then the Portable XRF measurements would have a significant bias because they would be accounting for extra-cerebral iron content.**

We have added this limitation to the Discussion section, acknowledging that while skin and scalp represent a relatively small proportion of the tissue mass/volume within the XRF beam, iron accumulation here cannot be excluded as a source of measurement bias.

8. **Please specify the DV coordinate of cell implantation in the brain because Figure 2A shows a high variability in the tumor growth zone.**

3.5 mm depth is now specified in the Methods section.

9. **The decision to administer radiotherapy to animals without clinical signs or imaging evidence of a tumor is confusing. Was the engraftment rate 100% in irradiated animals? What was the mean tumor size at the time of treatment? Have the authors used MRI to assess tumor growth kinetics and confirm tumor presence six days post-engraftment?**

Engraftment rate (100%) and median tumor volume at start of treatment (8 mm^3^) have been included in the Methods section. The justification for starting treatment on Day 6 was also based partly on our unpublished pilot studies assessing tumor growth kinetics in this model. In these pilot studies (N = 24), by day 6, we observed a 96% engraftment rate with a median tumor volume of 10 mm^3^. Growth kinetics for mice receiving chemoradiotherapy versus mice receiving no treatment diverged quickly after the start of treatment on day 6. Rapid tumor growth in the non-treated mice (median tumor volume of 43 mm^3^ on day 10) led to a median survival time of 13 days. In the irradiated mice, tumor growth was significantly delayed (median tumor volume of 13 mm^3^ on day 10) and then regressed in response to treatment (median tumor volume of 5 mm^3^ on day 34), leading to a median survival time of 59 days.

**Reviewer 2 comments**

1. **Considering biological complexity vs. calibration standards, the phantoms used for method validation do not fully replicate the biological complexity and inhomogeneity of real tissue, which can affect the accuracy and applicability of limit of detection calculations and linearity assessments in vivo. Can you please elaborate on this and provide some justifications?**

We thank the reviewer for bringing up this excellent point. We expanded this paragraph of the Discussion section to include more information about what the relevant biological complexity is and why it matters, as well as including some additional citations. This paragraph also emphasizes that we are calculating an effective limit of detection for each system rather than the true limit of detection for each method.

2. **The study was conducted in a murine glioblastoma model, which, while relevant, may not fully represent human brain tumors or other clinical scenarios. The number of biological replicates per group was relatively small (often 2–4), which may limit statistical power. Similarly, some methods, such as portable XRF, may not be directly translatable to human brain studies due to anatomical differences. Can you please add a paragraph with respect to translation of the results to clinical setting?**

We have added a paragraph in the Discussion section discussing potential applications of this work, including some limitations specific to clinical translation. We emphasize that this study establishes a starting point for future studies aiming for clinical translation, including the need for larger sample sizes in future work.

3. **With respect to the limitations in methodologies, a more in-depth discussion is required. For instance the upper limit of detection in MRI T2* mapping is limited at high iron concentrations. Doses above 100 mg/kg/day of ferumoxytol caused significant image distortion and signal dropout, making quantification unreliable at these levels. As such, influence of non-iron factors should be elaborated in more details.**

We have included further discussion as to the likely cause for signal dropout and expanded discussion of non-iron factors that influence T2*.

4. **The following statement is too optimistic, especially clinical part, please revise
   “This lends to the significance and impact of this work as these findings have implications in basic, preclinical, and clinical research investigating the impacts of iron concentrations on diverse aspects of human health and disease”**

We have amended this statement to reflect a more realistic view by removing the clinical impact clause and adding a statement that these results may provide guidance on future clinical studies. The revised statement now reads: “This lends to the significance and impact of this work as these findings have implications in basic and preclinical research investigating the impacts of iron concentrations on diverse aspects of human health and disease. These findings may also provide guidance for the design of future clinical studies assessing these iron-related topics.”

**Reviewer 3 comments:**

1. **It is strongly recommended that the authors compare their MRI data analysis approach with related publications, such as Roudi et al., 2024 in osteosarcoma.**

Our approach to MRI data analysis differs from that of Roudi et al. primarily only for image processing. We calculated T2* maps using in-house MATLAB code rather than using vendor-supplied software. We also utilized different software for manual delineation of tumor segments. In addition, Roudi et al., utilized masking to exclude tumor voxels with low signal intensity. However, the overarching workflow utilizing mono-exponential fitting of MGE scans to calculate T2* is the same. To address this comment, we have expanded the Methods section to provide additional citations employing the same software and workflows, and we have made our MATLAB code available as part of the manuscript supplementary materials.

2. **It has been shown that Fe is actively phagocytosed by macrophages; however, no data are provided regarding these cell populations.**

The reviewer brings up a good point, which we did not clearly articulate in the original submission. The in vitro study shown in Figure 4 is comparing ferumoxytol uptake in a glioma cell line and a macrophage cell line. We have expanded the text in this section to make this clearer in the revised submission.

3. **What is the rationale behind selecting T2* rather than T2?**

We have expanded our explanation for T2* and included an explanation for T2 in the Introduction, as well as rationale for why T2* was selected over T2.

**Journal/Editor comments**

1. **Please ensure that your manuscript meets PLOS ONE's style requirements, including those for file naming.**

We have reviewed PLOS ONE style requirements to make corrections to our manuscript formatting.

2. **Please note that PLOS One has specific guidelines on code sharing for submissions in which author-generated code underpins the findings in the manuscript. In these cases, we expect all author-generated code to be made available without restrictions upon publication of the work.**

Author-generated code that is critical to our findings has been made available in the Supporting Information section. This information has been added to our Data Availability Statement in the online form.

3. **Please include your full ethics statement in the ‘Methods’ section of your manuscript file.**

We have reviewed the ethics statement guidelines to ensure all required elements are included in our manuscript.

4. **All PLOS journals now require all data underlying the findings described in their manuscript to be freely available to other researchers, either 1. In a public repository, 2. Within the manuscript itself, or 3. Uploaded as supplementary information.**

We have included a file containing all relevant data in the Supporting Information section. This information has been added to our Data Availability Statement in the online form.

5. **We note that the grant information you provided in the ‘Funding Information’ and ‘Financial Disclosure’ sections do not match. Please ensure that you provide the correct grant numbers for the awards you received for your study in the ‘Funding Information’ section.**

We have corrected inconsistencies in our Financial Disclosure section of the online form. Please let us know if additional changes are necessary.

6. **Please state what role the funders took in the study. If the funders had no role, please state: "The funders had no role in study design, data collection and analysis, decision to publish, or preparation of the manuscript."**

We have now noted that the funders took no role in this study in our Financial Disclosure section.

7. **Please remove any funding-related text from the manuscript and let us know how you would like to update your Funding Statement.**

We removed mention of the NIH P30 grant supporting the Purdue Institute for Cancer Research from our acknowledgements section and ensured this information was included correctly in our Funding Disclosure.

8. **We note that Figure(s) 1, 3 in your submission contain copyrighted images… We require you to either (1) present written permission from the copyright holder to publish these figures specifically under the CC BY 4.0 license, or (2) remove the figures from your submission.**

Figure 1 has been modified to replace copyrighted icons with icons drawn by the authors. Figure 3.1 is an AI generated representation of a picture taken in our lab and is not copyrighted. A dedicated section has been added to the Methods portion of the manuscript to clearly describe how generative AI was used for creation of this diagram.

9. **If the reviewer comments include a recommendation to cite specific previously published works, please review and evaluate these publications to determine whether they are relevant and should be cited. There is no requirement to cite these works unless the editor has indicated otherwise.**

Reviewer 3 suggested that we compared our approach to analyzing our MRI data to other published methods like those used by Roudi et al., 2024. We have expanded our MRI methods to include this citation and additional citations which apply to our selected workflow.
