# Supplementary material for: Comparison of methods for in vivo and ex vivo quantification of iron in murine brain tumors
Source: PLoS One. 2026 Aug 17;21(8):e0356378. doi: 10.1371/journal.pone.0356378 (PMC13480596; doi:10.1371/journal.pone.0356378)
Supplement: Peer review history

# Author comment: Author response to Decision Letter 0

**DOI:** 10.1371/journal.pone.0356378.r001

**Transfer Alert**

This paper was transferred from another journal. As a result, its full editorial history (including decision letters, peer reviews and author responses) may not be present.

14 Apr 2026

# Peer review: Decision Letter 0

**DOI:** 10.1371/journal.pone.0356378.r002

**Authors:** Amitava Mukherjee

15 May 2026

Dear Dr. Scarpelli,

Thank you for submitting your manuscript to PLOS ONE. After careful consideration, we feel that it has merit but does not fully meet PLOS ONE’s publication criteria as it currently stands. Therefore, we invite you to submit a revised version of the manuscript that addresses the points raised during the review process.

Please submit your revised manuscript by Jun 29 2026 11:59PM. If you will need more time than this to complete your revisions, please reply to this message or contact the journal office at plosone@plos.org. When you're ready to submit your revision, log on to https://www.editorialmanager.com/pone/ and select the 'Submissions Needing Revision' folder to locate your manuscript file.

- A letter that responds to each point raised by the academic editor and reviewer(s). You should upload this letter as a separate file labeled 'Response to Reviewers'.
- A marked-up copy of your manuscript that highlights changes made to the original version. You should upload this as a separate file labeled 'Revised Manuscript with Track Changes'.
- An unmarked version of your revised paper without tracked changes. You should upload this as a separate file labeled 'Manuscript'.

If you would like to make changes to your financial disclosure, please include your updated statement in your cover letter. Guidelines for resubmitting your figure files are available below the reviewer comments at the end of this letter.

If applicable, we recommend that you deposit your laboratory protocols in protocols.io to enhance the reproducibility of your results. Protocols.io assigns your protocol its own identifier (DOI) so that it can be cited independently in the future. For instructions see: https://journals.plos.org/plosone/s/submission-guidelines#loc-laboratory-protocols. Additionally, PLOS ONE offers an option for publishing peer-reviewed Lab Protocol articles, which describe protocols hosted on protocols.io. Read more information on sharing protocols at https://plos.org/protocols?utm_medium=editorial-email&utm_source=authorletters&utm_campaign=protocols.

As the corresponding author, your ORCID iD is verified in the submission system and will appear in the published article. PLOS supports the use of ORCID, and we encourage all coauthors to register for an ORCID iD and use it as well. Please encourage your coauthors to verify their ORCID iD within the submission system before final acceptance, as unverified ORCID iDs will not appear in the published article. *Only*  the individual author can complete the verification step; PLOS staff *cannot*  verify ORCID iDs on behalf of authors.

We look forward to receiving your revised manuscript.

Kind regards,

Amitava Mukherjee, ME, Ph.D.

Academic Editor

PLOS One

Journal Requirements:

When submitting your revision, we need you to address these additional requirements.

1. Please ensure that your manuscript meets PLOS ONE's style requirements, including those for file naming. The PLOS ONE style templates can be found at

https://journals.plos.org/plosone/s/file?id=wjVg/PLOSOne_formatting_sample_main_body.pdf and https://journals.plos.org/plosone/s/file?id=ba62/PLOSOne_formatting_sample_title_authors_affiliations.pdf

2. Please note that PLOS One has specific guidelines on code sharing for submissions in which author-generated code underpins the findings in the manuscript. In these cases, we expect all author-generated code to be made available without restrictions upon publication of the work. Please review our guidelines at https://journals.plos.org/plosone/s/materials-and-software-sharing#loc-sharing-code and ensure that your code is shared in a way that follows best practice and facilitates reproducibility and reuse.

3. Please include your full ethics statement in the ‘Methods’ section of your manuscript file. In your statement, please include the full name of the IRB or ethics committee who approved or waived your study, as well as whether or not you obtained informed written or verbal consent. If consent was waived for your study, please include this information in your statement as well.

4. In the online submission form, you indicated that data are available upon request to the corresponding author.

All PLOS journals now require all data underlying the findings described in their manuscript to be freely available to other researchers, either 1. In a public repository, 2. Within the manuscript itself, or 3. Uploaded as supplementary information.

This policy applies to all data except where public deposition would breach compliance with the protocol approved by your research ethics board. If your data cannot be made publicly available for ethical or legal reasons (e.g., public availability would compromise patient privacy), please explain your reasons on resubmission and your exemption request will be escalated for approval.

5. We note that the grant information you provided in the ‘Funding Information’ and ‘Financial Disclosure’ sections do not match.

When you resubmit, please ensure that you provide the correct grant numbers for the awards you received for your study in the ‘Funding Information’ section.

6. Thank you for stating the following financial disclosure:

This work was supported by the National Institutes of Health (NIH/NINDS R01 NS131160 and

NIH/NCI P30 CA023168).

Please state what role the funders took in the study. If the funders had no role, please state: "The funders had no role in study design, data collection and analysis, decision to publish, or preparation of the manuscript."

If this statement is not correct you must amend it as needed.

Please include this amended Role of Funder statement in your cover letter; we will change the online submission form on your behalf.

7. Thank you for stating the following in the Acknowledgments Section of your manuscript:

The authors acknowledge the use of the Chemical Genomics Facility and the Small Animal MRI Facility, both of which are core facilities of the NIH-funded Indiana Clinical and Translational Sciences Institute. These cores are administered by the Purdue Institute for Drug Discovery and the Weldon School of Biomedical Engineering, respectively. ICP-MS samples were prepared and run by the Research Instrumentation Center in the Department of Chemistry at Purdue University. Murine intracranial injections were completed in partnership with the Biological Evaluation Shared Resource Facility under the Purdue Institute for Cancer Research, supported by P30 CA023168.

We note that you have provided funding information that is not currently declared in your Funding Statement. However, funding information should not appear in the Acknowledgments section or other areas of your manuscript. We will only publish funding information present in the Funding Statement section of the online submission form.

Please remove any funding-related text from the manuscript and let us know how you would like to update your Funding Statement. Currently, your Funding Statement reads as follows:

This work was supported by the National Institutes of Health (NIH/NINDS R01 NS131160 and

NIH/NCI P30 CA023168).

Please include your amended statements within your cover letter; we will change the online submission form on your behalf.

8. We note that Figure(s) 1, 3 in your submission contain copyrighted images. All PLOS content is published under the Creative Commons Attribution License (CC BY 4.0), which means that the manuscript, images, and Supporting Information files will be freely available online, and any third party is permitted to access, download, copy, distribute, and use these materials in any way, even commercially, with proper attribution. For more information, see our copyright guidelines: http://journals.plos.org/plosone/s/licenses-and-copyright.

We require you to either (1) present written permission from the copyright holder to publish these figures specifically under the CC BY 4.0 license, or (2) remove the figures from your submission:

a. You may seek permission from the original copyright holder of Figure(s) 1, 3 to publish the content specifically under the CC BY 4.0 license.

We recommend that you contact the original copyright holder with the Content Permission Form (http://journals.plos.org/plosone/s/file?id=7c09/content-permission-form.pdf) and the following text:

“I request permission for the open-access journal PLOS ONE to publish XXX under the Creative Commons Attribution License (CCAL) CC BY 4.0 (http://creativecommons.org/licenses/by/4.0/). Please be aware that this license allows unrestricted use and distribution, even commercially, by third parties. Please reply and provide explicit written permission to publish XXX under a CC BY license and complete the attached form.”

Please upload the completed Content Permission Form or other proof of granted permissions as an "Other" file with your submission.

In the figure caption of the copyrighted figure, please include the following text: “Reprinted from [ref] under a CC BY license, with permission from [name of publisher], original copyright [original copyright year].”

b. If you are unable to obtain permission from the original copyright holder to publish these figures under the CC BY 4.0 license or if the copyright holder’s requirements are incompatible with the CC BY 4.0 license, please either i) remove the figure or ii) supply a replacement figure that complies with the CC BY 4.0 license. Please check copyright information on all replacement figures and update the figure caption with source information. If applicable, please specify in the figure caption text when a figure is similar but not identical to the original image and is therefore for illustrative purposes only.

9. If the reviewer comments include a recommendation to cite specific previously published works, please review and evaluate these publications to determine whether they are relevant and should be cited. There is no requirement to cite these works unless the editor has indicated otherwise.

[Note: HTML markup is below. Please do not edit.]

Reviewers' comments:

Reviewer's Responses to Questions

**Comments to the Author**

1. Is the manuscript technically sound, and do the data support the conclusions?

Reviewer #1: Partly

Reviewer #2: Yes

Reviewer #3: Partly

**********

2. Has the statistical analysis been performed appropriately and rigorously?

Reviewer #1: N/A

Reviewer #2: Yes

Reviewer #3: Yes

**********

3. Have the authors made all data underlying the findings in their manuscript fully available?

The PLOS Data policy

Reviewer #1: Yes

Reviewer #2: Yes

Reviewer #3: Yes

**********

4. Is the manuscript presented in an intelligible fashion and written in standard English?

Reviewer #1: Yes

Reviewer #2: Yes

Reviewer #3: Yes

**********

Reviewer #1: In this manuscript, the authors have focused on analyzing multiple methods for quantifying iron oxide nanoparticles across in vivo, ex vivo, and in vitro models of glioma or glioblastoma. The topic is timely and relevant; however, some points need to be reviewed.

After reading the introduction, the rationale for administering ferumoxytol in the different models remains unclear. The authors mention that this drug is administered as an MRI contrast agent. What is the incidence of this drug's use as a contrast agent in clinical practice?

What is the significance of detecting elevated iron levels induced by ferumoxytol when iron levels in the brain are actually very low? If I understand correctly, the nanoparticle is primarily a tool for validating and standardizing iron quantification. If so, please specify this clearly in the text.

The dosing scheme (up to 300 mg/kg/day × 21 injections) is extremely high relative to clinical use of ferumoxytol. What is the biological relevance to human imaging or iron physiology? Therefore, it is recommended to clearly justify the selection of doses and, based on that, discuss the clinical translation of the results.

The authors mention that a descending trend was also observed in mice receiving 0 mg/kg ferumoxytol. This appears to be critical because it suggests that changes in T2 are not specific to iron accumulation. What factors in tumor progression might be influencing this signal? Histological validation would be valuable to elucidate this result.

What is the probability that in vivo analysis with the Portable XRF is dependent on the position of the beam and the rodent's head?

Is it likely that iron will accumulate in the hair and scalp after ferumoxytol administration? If so, then the Portable XRF measurements would have a significant bias because they would be accounting for extra-cerebral iron content.

Please specify the DV coordinate of cell implantation in the brain because Figure 2A shows a high variability in the tumor growth zone.

The decision to administer radiotherapy to animals without clinical signs or imaging evidence of a tumor is confusing. Was the engraftment rate 100% in irradiated animals? What was the mean tumor size at the time of treatment? Have the authors used MRI to assess tumor growth kinetics and confirm tumor presence six days post-engraftment?

Reviewer #2: The manuscript by Veenstrata et al., reads clearly, coherently and with a strong scientific narrative except for the discussion section. The following issues should be addressed before it can be suitable for publication in PLOS One.

Considering biological complexity vs. calibration standards, the phantoms used for method validation do not fully replicate the biological complexity and inhomogeneity of real tissue, which can affect the accuracy and applicability of limit of detection calculations and linearity assessments in vivo. Can you please elaborate on this and provide some justifications?

The study was conducted in a murine glioblastoma model, which, while relevant, may not fully represent human brain tumors or other clinical scenarios. The number of biological replicates per group was relatively small (often 2–4), which may limit statistical power. Similarly, some methods, such as portable XRF, may not be directly translatable to human brain studies due to anatomical differences. Can you please add a paragraph with respect to translation of the results to clinical setting?

With respect to the limitations in methodologies, a more in-depth discussion is required. For instance the upper limit of detection in MRI T2* mapping is limited at high iron concentrations. Doses above 100 mg/kg/day of ferumoxytol caused significant image distortion and signal dropout, making quantification unreliable at these levels. As such, influence of non-iron factors should be elaborated in more details.

The following statement is too optimistic, especially clinical part, please revise

“This lends to the significance and impact of this work as these findings have implications in basic, preclinical, and clinical research investigating the impacts of iron concentrations on diverse aspects of human health and disease”

Reviewer #3: 1) It is strongly recommended that the authors compare their MRI data analysis approach with related publications, such as Roudi et al., 2024 in osteosarcoma.

2) It has been shown that Fe is actively phagocytosed by macrophages; however, no data are provided regarding these cell populations.

3) What is the rationale behind selecting T2* rather than T2?

**********

what does this mean?). If published, this will include your full peer review and any attached files.

If you choose “no”, your identity will remain anonymous but your review may still be made public.

**Do you want your identity to be public for this peer review?**  For information about this choice, including consent withdrawal, please see our Privacy Policy

Reviewer #1: No

Reviewer #2: No

Reviewer #3: No

**********

[NOTE: If reviewer comments were submitted as an attachment file, they will be attached to this email and accessible via the submission site. Please log into your account, locate the manuscript record, and check for the action link "View Attachments". If this link does not appear, there are no attachment files.]

To ensure your figures meet our technical requirements, please review our figure guidelines: https://journals.plos.org/plosone/s/figures

You may also use PLOS’s free figure tool, NAAS, to help you prepare publication quality figures: https://journals.plos.org/plosone/s/figures#loc-tools-for-figure-preparation.

NAAS will assess whether your figures meet our technical requirements by comparing each figure against our figure specifications.

# Author comment: Author response to Decision Letter 1

**DOI:** 10.1371/journal.pone.0356378.r003

9 Jul 2026

Response to comments

The authors greatly appreciate the time and effort of the editor and the reviewers to assess our manuscript and give valuable feedback. We have taken each comment into careful consideration and modified the manuscript accordingly. Please see responses to individual comments below.

Reviewer 1 comments

1. After reading the introduction, the rationale for administering ferumoxytol in the different models remains unclear.

We agree that this rationale was underdeveloped in the manuscript. We have addressed this by expanding on the theragnostic potential of ferumoxytol and discussing the importance of quantification for monitoring disease progression and therapeutic concentrations (please see the comments in the revised manuscript, which indicate the specific sections that were added/altered to address each comment).

2. The authors mention that this drug is administered as an MRI contrast agent. What is the incidence of this drug's use as a contrast agent in clinical practice?

Although the total incidence of ferumoxytol’s off-label use as an MRI contrast agent is difficult to determine, we have now included manufacturer-reported incidence values for all ferumoxytol use (2.6 million uses), as well as incidence of ferumoxytol use as an MRI contrast agent in a multicenter safety analysis (4000+ uses over a 15-year period across six centers).

3. What is the significance of detecting elevated iron levels induced by ferumoxytol when iron levels in the brain are actually very low? If I understand correctly, the nanoparticle is primarily a tool for validating and standardizing iron quantification. If so, please specify this clearly in the text.

We added a sentence to the Introduction about our model being an ideal system for developing, testing, and comparing our methods for iron quantification. We believe our additions to address comment #1 also help to address this comment. Ferumoxytol is being tested as a therapeutic agent, so the methods developed here are geared towards assessing iron in those applications or diseases where iron concentrations might be higher than normal brain levels e.g. brain tumor.

4. The dosing scheme (up to 300 mg/kg/day × 21 injections) is extremely high relative to clinical use of ferumoxytol. What is the biological relevance to human imaging or iron physiology? Therefore, it is recommended to clearly justify the selection of doses and, based on that, discuss the clinical translation of the results.

We agree, these doses are relatively high compared to current human usage. This is for multiple reasons, including 1) our use of ferumoxytol as a cancer therapeutic, which requires higher doses than current applications and 2) the differences in body surface area between mice and humans. We have included a brief mention of the dose range when converted to human equivalent dose based on organism surface area to help in part account for faster metabolism of the drug in mice (using guidance from the FDA). This is partly related to the short plasma half-life of ferumoxytol in mice compared to humans, which has also been noted in the revised manuscript. Once converted to the human equivalent dose, the tested dose ranges in this study become more clinically relevant.

5. The authors mention that a descending trend was also observed in mice receiving 0 mg/kg ferumoxytol. This appears to be critical because it suggests that changes in T2 are not specific to iron accumulation. What factors in tumor progression might be influencing this signal? Histological validation would be valuable to elucidate this result.

We have clarified some potential non-iron factors that could contribute to decreasing T2* in the control mice in the Discussion and have included references for other studies, which document these effects. Unfortunately, histological validation was not possible as samples were destroyed for further iron analyses; however, some of the aforementioned references do include pathologic correlations with T2*.

6. What is the probability that in vivo analysis with the Portable XRF is dependent on the position of the beam and the rodent's head?

This is an important point, since the position of the beam on the rodent’s head will affect the Portable XRF output. To mitigate positional uncertainties, we developed a strategy to ensure reproducible placement of the Portable XRF. We have emphasized in the Methods section that we used this positioning strategy to minimize variation in beam placement and therefore any associated variation in measurement output. We have also added this as a potential source of uncertainty to the Portable XRF measurements in the Discussion section of the revised manuscript.

7. Is it likely that iron will accumulate in the hair and scalp after ferumoxytol administration? If so, then the Portable XRF measurements would have a significant bias because they would be accounting for extra-cerebral iron content.

We have added this limitation to the Discussion section, acknowledging that while skin and scalp represent a relatively small proportion of the tissue mass/volume within the XRF beam, iron accumulation here cannot be excluded as a source of measurement bias.

8. Please specify the DV coordinate of cell implantation in the brain because Figure 2A shows a high variability in the tumor growth zone.

3.5 mm depth is now specified in the Methods section.

9. The decision to administer radiotherapy to animals without clinical signs or imaging evidence of a tumor is confusing. Was the engraftment rate 100% in irradiated animals? What was the mean tumor size at the time of treatment? Have the authors used MRI to assess tumor growth kinetics and confirm tumor presence six days post-engraftment?

Engraftment rate (100%) and median tumor volume at start of treatment (8 mm3) have been included in the Methods section. The justification for starting treatment on Day 6 was also based partly on our unpublished pilot studies assessing tumor growth kinetics in this model. In these pilot studies (N = 24), by day 6, we observed a 96% engraftment rate with a median tumor volume of 10 mm3. Growth kinetics for mice receiving chemoradiotherapy versus mice receiving no treatment diverged quickly after the start of treatment on day 6. Rapid tumor growth in the non-treated mice (median tumor volume of 43 mm3 on day 10) led to a median survival time of 13 days. In the irradiated mice, tumor growth was significantly delayed (median tumor volume of 13 mm3 on day 10) and then regressed in response to treatment (median tumor volume of 5 mm3 on day 34), leading to a median survival time of 59 days.

Reviewer 2 comments

1. Considering biological complexity vs. calibration standards, the phantoms used for method validation do not fully replicate the biological complexity and inhomogeneity of real tissue, which can affect the accuracy and applicability of limit of detection calculations and linearity assessments in vivo. Can you please elaborate on this and provide some justifications?

We thank the reviewer for bringing up this excellent point. We expanded this paragraph of the Discussion section to include more information about what the relevant biological complexity is and why it matters, as well as including some additional citations. This paragraph also emphasizes that we are calculating an effective limit of detection for each system rather than the true limit of detection for each method.

2. The study was conducted in a murine glioblastoma model, which, while relevant, may not fully represent human brain tumors or other clinical scenarios. The number of biological replicates per group was relatively small (often 2–4), which may limit statistical power. Similarly, some methods, such as portable XRF, may not be directly translatable to human brain studies due to anatomical differences. Can you please add a paragraph with respect to translation of the results to clinical setting?

We have added a paragraph in the Discussion section discussing potential applications of this work, including some limitations specific to clinical translation. We emphasize that this study establishes a starting point for future studies aiming for clinical translation, including the need for larger sample sizes in future work.

3. With respect to the limitations in methodologies, a more in-depth discussion is required. For instance the upper limit of detection in MRI T2* mapping is limited at high iron concentrations. Doses above 100 mg/kg/day of ferumoxytol caused significant image distortion and signal dropout, making quantification unreliable at these levels. As such, influence of non-iron factors should be elaborated in more details.

We have included further discussion as to the likely cause for signal dropout and expanded discussion of non-iron factors that influence T2*.

4. The following statement is too optimistic, especially clinical part, please revise

“This lends to the significance and impact of this work as these findings have implications in basic, preclinical, and clinical research investigating the impacts of iron concentrations on diverse aspects of human health and disease”

We have amended this statement to reflect a more realistic view by removing the clinical impact clause and adding a statement that these results may provide guidance on future clinical studies. The revised statement now reads: “This lends to the significance and impact of this work as these findings have implications in basic and preclinical research investigating the impacts of iron concentrations on diverse aspects of human health and disease. These findings may also provide guidance for the design of future clinical studies assessing these iron-related topics.” Reviewer 3 comments:

Reviewer 3 comments:

1. It is strongly recommended that the authors compare their MRI data analysis approach with related publications, such as Roudi et al., 2024 in osteosarcoma.

Our approach to MRI data analysis differs from that of Roudi et al. primarily only for image processing. We calculated T2* maps using in-house MATLAB code rather than using vendor-supplied software. We also utilized different software for manual delineation of tumor segments. In addition, Roudi et al., utilized masking to exclude tumor voxels with low signal intensity. However, the overarching workflow utilizing mono-exponential fitting of MGE scans to calculate T2* is the same. To address this comment, we have expanded the Methods section to provide additional citations employing the same software and workflows, and we have made our MATLAB code available as part of the manuscript supplementary materials.

2. It has been shown that Fe is actively phagocytosed by macrophages; however, no data are provided regarding these cell populations.

The reviewer brings up a good point, which we did not clearly articulate in the original submission. The in vitro study shown in Figure 4 is comparing ferumoxytol uptake in a glioma cell line and a macrophage cell line. We have expanded the text in this section to make this clearer in the revised submission.

3. What is the rationale behind selecting T2* rather than T2?

We have expanded our explanation for T2* and included an explanation for T2 in the Introduction, as well as rationale for why T2* was selected over T2.

Journal/Editor comments

1. Please ensure that your manuscript meets PLOS ONE's style requirements, including those for file naming.

We have reviewed PLOS ONE style requirements to make corrections to our manuscript formatting.

2. Please note that PLOS One has specific guidelines on code sharing for submissions in which author-generated code underpins the findings in the manuscript. In these cases, we expect all author-generated code to be made available without restrictions upon publication of the work.

Author-generated code that is critical to our findings has been made available in the Supporting Information section. This information has been added to our Data Availability Statement in the online form.

3. Please include your full ethics statement in the ‘Methods’ section of your manuscript file.

We have reviewed the ethics statement guidelines to ensure all required elements are included in our manuscript.

4. All PLOS journals now require all data underlying the findings described in their manuscript to be freely available to other researchers, either 1. In a public repository, 2. Within the manuscript itself, or 3. Uploaded as supplementary information.

We have included a file containing all relevant data in the Supporting Information section. This information has been added to our Data Availability Statement in the online form.

5. We note that the grant information you provided in the ‘Funding Information’ and ‘Financial Disclosure’ sections do not match. Please ensure that you provide the correct grant numbers for the awards you received for your study in the ‘Funding Information’ section.

We have corrected inconsistencies in our Financial Disclosure section of the online form. Please let us know if additional changes are necessary.

6. Please state what role the funders took in the study. If the funders had no role, please state: "The funders had no role in study design, data collection and analysis, decision to publish, or preparation of the manuscript."

We have now noted that the funders took no role in this study in our Financial Disclosure section.

7. Please remove any funding-related text from the manuscript and let us know how you would like to update your Funding Statement.

We removed mention of the NIH P30 grant supporting the Purdue Institute for Cancer Research from our acknowledgements section and ensured this information was included correctly in our Funding Disclosure.

8. We note that Figure(s) 1, 3 in your submission contain copyrighted images… We require you to either (1) present written permission from the copyright holder to publish these figures specifically under the CC BY 4.0 license, or (2) remove the figures from your submission.

Figure 1 has been modified to replace copyrighted icons with icons drawn by the authors. Figure 3.1 is an AI generated representation of a picture taken in our lab and is not copyrighted. A dedicated section has been added to the Methods portion of the manuscript to clearly describe how generative AI was used for creation of this diagram.

9. If the reviewer comments include a recommendation to cite specific previously published works, please review and evaluate these publications to determine whether they are relevant and should be cited. There is no requirement to cite these works unless the editor has indicated otherwise.

Reviewer 3 suggested that we compared our approach to analyzing our MRI data to other published methods like those used by Roudi et al., 2024. We have expanded our MRI methods to include this citation and additional citations which apply to our selected workflow.

Attachment Submitted filename: Response to Reviewers.docx

# Peer review: Decision Letter 1

**DOI:** 10.1371/journal.pone.0356378.r004

**Authors:** Amitava Mukherjee

3 Aug 2026

Comparison of methods for in vivo and ex vivo quantification of iron in murine brain tumors

PONE-D-26-14543R1

Dear Dr. Scarpelli,

We’re pleased to inform you that your manuscript has been judged scientifically suitable for publication and will be formally accepted for publication once it meets all outstanding technical requirements.

Within one week, you’ll receive an e-mail detailing the required amendments. When these have been addressed, you’ll receive a formal acceptance letter and your manuscript will be scheduled for publication.

An invoice will be generated when your article is formally accepted. Please note, if your institution has a publishing partnership with PLOS and your article meets the relevant criteria, all or part of your publication costs will be covered. Please make sure your user information is up-to-date by logging into Editorial Manager at Editorial Manager® and clicking the ‘Update My Information' link at the top of the page. For questions related to billing, please contact billing support.

If your institution or institutions have a press office, please notify them about your upcoming paper to help maximize its impact. If they’ll be preparing press materials, please inform our press team as soon as possible -- no later than 48 hours after receiving the formal acceptance. Your manuscript will remain under strict press embargo until 2 pm Eastern Time on the date of publication. For more information, please contact onepress@plos.org.

Kind regards,

Amitava Mukherjee, ME, Ph.D.

Academic Editor

PLOS One

Additional Editor Comments (optional):

Reviewers' comments:

Reviewer's Responses to Questions

**Comments to the Author**

Reviewer #1: All comments have been addressed

Reviewer #2: All comments have been addressed

Reviewer #3: (No Response)

**********

2. Is the manuscript technically sound, and do the data support the conclusions?

Reviewer #1: Yes

Reviewer #2: Partly

Reviewer #3: (No Response)

**********

3. Has the statistical analysis been performed appropriately and rigorously?

Reviewer #1: Yes

Reviewer #2: Yes

Reviewer #3: (No Response)

**********

4. Have the authors made all data underlying the findings in their manuscript fully available?

The PLOS Data policy

Reviewer #1: Yes

Reviewer #2: Yes

Reviewer #3: (No Response)

**********

5. Is the manuscript presented in an intelligible fashion and written in standard English?

Reviewer #1: Yes

Reviewer #2: Yes

Reviewer #3: (No Response)

**********

Reviewer #1: The authors have satisfactorily addressed all observations. The revised manuscript has been considerably improved and can therefore be published in its current form.

Reviewer #2: All the raised comments have been addressed by the authors. However, the high doses of the drug remains as a limitation with respect to translation of the data to the clinical setting

Reviewer #3: The authors addressed the comments and I have no further comments. The manuscript can be published in the current format.

**********

what does this mean?). If published, this will include your full peer review and any attached files.

If you choose “no”, your identity will remain anonymous but your review may still be made public.

**Do you want your identity to be public for this peer review?**  For information about this choice, including consent withdrawal, please see our Privacy Policy

Reviewer #1: No

Reviewer #2: No

Reviewer #3: No

**********

# Editor report: Acceptance letter

**DOI:** 10.1371/journal.pone.0356378.r005

**Authors:** Amitava Mukherjee

PONE-D-26-14543R1

PLOS One

Dear Dr. Scarpelli,

I'm pleased to inform you that your manuscript has been deemed suitable for publication in PLOS One. Congratulations! Your manuscript is now being handed over to our production team.

At this stage, our production department will prepare your paper for publication. This includes ensuring the following:

* All references, tables, and figures are properly cited

* All relevant supporting information is included in the manuscript submission,

* There are no issues that prevent the paper from being properly typeset

You will receive further instructions from the production team, including instructions on how to review your proof when it is ready. Please keep in mind that we are working through a large volume of accepted articles, so please give us a few days to review your paper and let you know the next and final steps.

Lastly, if your institution or institutions have a press office, please let them know about your upcoming paper now to help maximize its impact. If they'll be preparing press materials, please inform our press team within the next 48 hours. Your manuscript will remain under strict press embargo until 2 pm Eastern Time on the date of publication. For more information, please contact onepress@plos.org.

You will receive an invoice from PLOS for your publication fee after your manuscript has reached the completed accept phase. If you receive an email requesting payment before acceptance or for any other service, this may be a phishing scheme. Learn how to identify phishing emails and protect your accounts at https://explore.plos.org/phishing.

If we can help with anything else, please email us at customercare@plos.org.

Thank you for submitting your work to PLOS ONE and supporting open access.

Kind regards,

PLOS ONE Editorial Office Staff

on behalf of

Professor Dr. Amitava Mukherjee

Academic Editor

PLOS One
